# Review: Emerging Eye-Based Diagnostic Technologies for Traumatic Brain Injury

**DOI:** 10.1109/RBME.2022.3161352

**Published:** 2022-03-23

**Authors:** Georgia Harris, Jonathan James Stanley Rickard, Gibran Butt, Liam Kelleher, Richard James Blanch, Jonathan Cooper, Pola Goldberg Oppenheimer

**Affiliations:** School of Chemical Engineering, Advanced Nanomaterials Structures and Applications Laboratories, College of Engineering and Physical SciencesUniversity of Birmingham 1724 B15 2TT Birmingham U.K.; Department of Physics, Cavendish LaboratoryUniversity of Cambridge 2152 CB3 0HE Cambridge U.K.; School of Chemical Engineering, Advanced Nanomaterials Structures and Applications Laboratories, College of Engineering and Physical SciencesUniversity of Birmingham 1724 B15 2TT Birmingham U.K.; School of Chemical Engineering, Advanced Nanomaterials Structures and Applications Laboratories, College of Engineering and Physical SciencesUniversity of Birmingham 1724 B15 2TT Birmingham U.K.; Healthcare Technologies Institute, Institute of Translational Medicine 572804 B15 2TH Birmingham U.K.; Ophthalmology DepartmentUniversity Hospitals Birmingham NHS Foundation Trust 1732 B15 2TH Birmingham U.K.; Department of Military Surgery and TraumaRoyal Centre for Defence Medicine 101807 B15 2TH Birmingham U.K.; Neuroscience and Ophthalmology, Department of Ophthalmology, University Hospitals Birmingham NHS Foundation Trustc 1732 Birmingham U.K.; School of Biomedical EngineeringUniversity of Glasgow 3526 G12 8LT Glasgow U.K.

**Keywords:** Biomedical engineering, biomedical optical imaging, biophotonics, biosensors, medical devices, molecular imaging, neurology, ophthalmology, optic nerve, optical sensors, point of care, raman scattering, retina, traumatic brain injury (TBI)

## Abstract

The study of ocular manifestations of neurodegenerative disorders, *Oculomics,* is a growing field of investigation for early diagnostics, enabling structural and chemical biomarkers to be monitored overtime to predict prognosis. Traumatic brain injury (TBI) triggers a cascade of events harmful to the brain, which can lead to neurodegeneration. TBI, termed the “silent epidemic” is becoming a leading cause of death and disability worldwide. There is currently no effective diagnostic tool for TBI, and yet, early-intervention is known to considerably shorten hospital stays, improve outcomes, fasten neurological recovery and lower mortality rates, highlighting the unmet need for techniques capable of rapid and accurate point-of-care diagnostics, implemented in the earliest stages. This review focuses on the latest advances in the main neuropathophysiological responses and the achievements and shortfalls of TBI diagnostic methods. Validated and emerging TBI-indicative biomarkers are outlined and linked to ocular neuro-disorders. Methods detecting structural and chemical ocular responses to TBI are categorised along with prospective chemical and physical sensing techniques. Particular attention is drawn to the potential of Raman spectroscopy as a non-invasive sensing of neurological molecular signatures in the ocular projections of the brain, laying the platform for the first tangible path towards alternative point-of-care diagnostic technologies for TBI

## I. Introduction

Traumatic Brain Injury (TBI) can cause death or lifelong physical and mental disability. TBI occurs when the brain is damaged by rapid acceleration or deceleration with rotational or shear forces or penetration [1]. It is estimated that between 54 - 69 million TBI occur worldwide each year [2]–[5]. In the U.K. specifically, head injuries are the leading cause of death in the under 40-year-olds with around 1.4 million emergency departments episodes every year [6], [7]. If the patient survives the TBI there can be long term socioeconomic costs related to lost economic output and a requirement for care caused by the permanent brain damage. Not everyone who suffers trauma to the head has significant TBI, but both diagnosis and distinguishing TBI severity can be very challenging. The most global common causes of TBI are car accidents, falls and assaults [8]–[12], and the most frequent victims of TBI are infants (0-4 years), young adults (15- 25 years) and the elderly (65+ years) [13], [14]. This is true in both high and low-to-middle income countries (LMIC), though the exact numbers and cases are uncertain as data concerning TBI in LMICs are scarce, despite it being a prevalent public health issue [12], [15]–[18]. For instance, a Nigerian study, at the Lagos State University Teaching Hospital in Ikeja, found that 23% of TBI patients were referred to non-trauma centres because of a lack of bed space [12]. Hospital bed spaces are scarce in LMICs, but also in high income countries and may be occupied by patients misdiagnosed with TBI or with severity over-diagnosed, both of which could be avoided by improved triage [19]. Triaging can also prevent unnecessary time and costs spent on healthcare services used for scanning and monitoring.

While there are numerous extensive reviews of ocular manifestations of common neurodegenerative (ND) diseases [20]–[28], changes in the visual system after TBI, a common neurological condition with huge and growing socioeconomic implications is significantly less well reviewed [29]–[32]. TBI increases rates of ND disorders [33], [34], as trauma triggers neurodegeneration accompanied by an increase in ND biomarkers, such as amyloid-Beta and tau protein [29], [35]–[37]. This review summarises the strengths and weaknesses of current TBI diagnostic approaches and the need for new developments in biochemical diagnostic techniques, which are non-invasive and can be implemented in the acute phase of brain injury. Building on the similarities in function and responses between the central nervous and the visual systems [38], along with the existing evidence of ocular changes associated with neurodegeneration creates a strong foundation for identifying TBI through its retinal and optic nerve (ON) manifestations.

### A. Traumatic Brain Injury

**Fig. 1.**
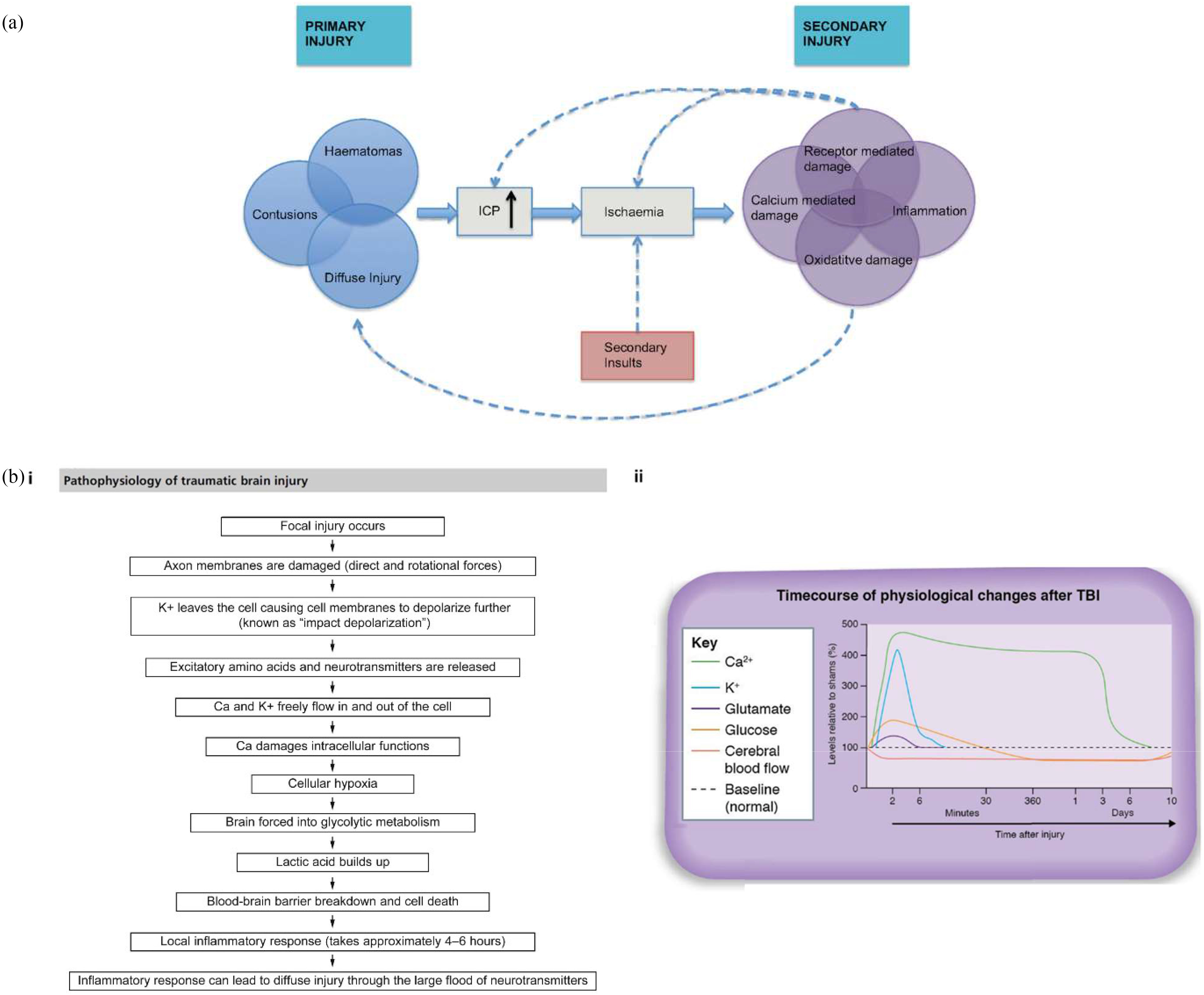
(a) Inter-relationship between the primary and secondary injuries of TBI. Secondary injuries can contribute to the initial primary ones, creating a cycle causing further damage [43]. (bi) Flowchart of the pathophysiological responses to TBI at a cellular level, reproduced with permission from [1] (ii) Timescale of neurochemical and metabolic changes that take place following moderate to severe TBI [11].

TBI may occur as a result of closed, open or crush head injuries, disturbing brain function. Most commonly, there is no break to the skin and the brain accelerates and/or decelerates within the closed skull, twisting, stretching and damaging the axons and blood vessels. Less often, the brain is exposed by an open injury whilst the skull base and brain stem may be damaged by crush injury [39]. Initial trauma causes a primary injury where the physical impact damages the cranial structures, then pathophysiological consequences of the trauma lead to secondary injuries and neurodegenerative processes [40], [41]. Acute axonal and nerve soma damage will usually cause concussion or coma, with duration depending on injury severity, and loss of function such as limb movement, speech, and executive and emotional impairments related to the injury location [7], [17], [39], [42]. This is not always clearly stratified, as the magnitude of biochemical reactions taking place following the primary injury, both contributing to and worsening the patient's state, is not known and its role in initiating a cycle of neurodegeneration is unclear (Fig. 1(a)) [43].

The most common brain areas involved in TBI are frontal and temporal lobes [44]. TBI may be categorised as mild, moderate, or severe, although symptom severity does not always coincide with injury severity, making TBI severity difficult to diagnose and manage. Mild TBI develops after stretching of neuronal plasma membranes and has common symptoms of headaches, dizziness, nausea, confusion and disorientation, which can last over hours, days or weeks [11], [45]–[47]. More specific inclusion criteria for mild TBI includes loss of consciousness for 30 minutes or less and/or post-traumatic amnesia for less than 24 hours [48].

Moderate to severe TBI as associated with hematomas (blood leakage) and immediate tissue death, not only losing brain functionality but also releasing toxins [45]. Most of these injuries result in unconsciousness for over 6 hours and long-term effects such as, cognitive and behavioural deficits, often resulting in aggressive behaviour, balance issues, disorientation or memory problems [46], [49]. After TBI, a progressive deterioration of grey and white brain matter can continue for up to a year post-trauma [17]. Sustaining a TBI of any severity can have long-term, detrimental, neurological effects, affecting patient's mobility, cognitive function, social and employable capabilities, economic situation and overall quality of life [7], [17], [50].

TBI has been termed a “silent epidemic”, as many incidents go unreported by patients or unrecognised by healthcare professionals [1], [51], thus accurate and reliable reports regarding pathology and costs associated with head injury are limited [52]. Whilst Road Traffic Accidents (RTAs) contribute to 60% of total TBI burden, and make up 2.5% of total deaths worldwide [53], [54]; TBI is also one of the most common injuries sustained by military personnel, with over 400000 incidents between 2000 and 2018 recorded in the U.S. Army alone [55]. Most TBI in service personnel are non-battle injuries, sustained during training and motor vehicle accidents [55]. Members of the Armed Forces on deployment are susceptible to enemy action as well as non-battle injury mechanisms and exposure to blast waves [56], [57]. Battle injuries are most commonly blast related TBI and are also frequently sustained by civilians who comprise the most common casualties in modern warfare [57], [58]. TBI is also prevalent in collision sports, American football and football players are at high risk of developing chronic traumatic encephalopathy (CTE) following impact with other players and “heading” balls [59]. In all the aforementioned contexts, TBI sufferers would benefit from point-of-care (POC) diagnostic techniques implemented outside of hospital settings to start the patient journey roadside, pitch-side and in hostile environments.

### B. Current ‘State-of-the-Art’ Diagnosis of Traumatic Brain Injury and the Unmet Need

TBI pathophysiology can be split into acute, sub-acute and chronic phases, occurring within 3-24 hours, 1 day - 3 weeks and from 3 weeks of the trauma, respectively [40], [60]. Fast, accurate and accessible diagnostics are critical for good TBI patient outcomes as secondary injuries such as hypoxia and inflammation develop in the acute period (≤ 1hour) after primary injury [61]. These pathologies account for much TBI-related morbidity and mortality (Fig. 1(bi)) [1], [62], and sorting TBI triage into either mild, moderate or severe categories in the acute phase will ensure the patient receives appropriate initial access to neurosurgical care and later neurorehabilitation [63].

Most established TBI diagnostic techniques can only be implemented in hospital, and a patient would typically undergo multiple examinations before a final diagnosis is made. Out-of-hospital assessment of TBI severity includes the Glasgow Coma Scale (GCS), a neurological scoring system of the patient's conscious state. Neuroimaging is usually only available in a hospital setting, as are surgical interventions such as decompression and insertion of monitoring devices [64]. Many methods take single time-point measurements, such as neuro-imaging, which requires intra-hospital transport and is therefore limited in frequency in severe TBI because transporting patients out of ICU increases risk of adverse effects, especially in those requiring continuous sedation in addition to the risks of ionising radiation exposure [65]. Single time-point measurements are unable to capture the dynamically changing state of the patient and can result in misleading diagnoses [66]. An ‘ideal’ method would monitor continuously or frequently without being laborious or causing harm.

#### 1) Glasgow Coma Scale

The National Institute for Health and Care Excellence (NICE) highlighted the need for a test to allow paramedics to determine if the patient should be transported from the scene of injury directly to the nearest neurological centre or a hospital with particular resuscitation resources [7]. In current practice, a clinician assesses TBI severity using the GCS, introduced in 1974 [67], which evaluates severity and predicts outcome using three main indicators, eye opening, motor response and verbal activity. The visual, verbal and motor responses give an indication as to which regions of the brain are damaged [68]. The GCS was developed as a fast, simple, bedside method to categorise mild to severe TBI by assessing the patient's level of consciousness, with categories being mild (13-15), moderate (9-12) and severe (3-8) [42], [69]. The GCS is widely used in over 80 countries [69], but has a number of limitations. For instance, a study showed that when two physicians independently assessed the same TBI patient using the GCS score within a 5-minute window there was a disagreement in 68% of cases by at least one sub-score [70]. In addition, in the common scenario of TBI sustained under the influence of alcohol or other drugs, intoxication limits the utility of this cognitive assessment especially, during the acute period before the patient is stabilised [42], [71], [72]. Moreover, the GCS system is not effective for TBI patients who are not capable of completing all three assessment sections [73], which typically occurs in cases of sedation, intubation, intoxication, pre-existing neurological diseases (such as pre-existing dementia [74]), disabilities (such as deafness, blindness, and paralysis), language barriers and infancy [7], [72], [75]. Such incompleteness inevitably skews the final score and predictive value, especially as the GCS relies heavily on the motor response section and makes interventions difficult to implement correctly in short timeframes, which are crucial for survival [71].

Even in the absence of confounding factors, the prognostic accuracy of the on-site GCS assessment is lower than GCS on admission to hospital [71], with admission scores, when the patient is more stable, being more accurate predictors of outcome. The acute, pre-hospital, period, however, is a vital time for diagnosis and clinical decisions made here, in the “golden hour”, for management and treatment have disproportionate influence on outcomes. Whilst it is the current gold standard, GCS is therefore limited in utility as a predictor of outcome in the pre-hospital space by its subjectivity, low inter-observer agreement and instability-related inaccuracy. However, the GCS is the only non-invasive diagnostic tool that is capable of assessing severity throughout the entire patient journey, and despite negative aspects, the GCS has been shown to correlate with more recent research in TBI diagnostics using biomarkers, neuroimaging and metabolomics [69], and it is commonly used to stratify patients for neuroimaging [76]. The validity of the GCS has been tested during the many years of use against the pathophysiology of TBI, with low scores being associated with lower cerebral metabolic rates, raised intracranial pressure (ICP) and abnormalities detected through neuroimaging [10], [11], [77], [78]. Low GCS scores are also reported alongside increased concentrations of blood biomarkers denoting trauma [69], [79].

#### 2) Other Diagnostic Tests

Other current early diagnostic techniques include neuroimaging and ICP monitoring, which are often equivocal on the presence and severity of TBI, particularly in the mild and moderate groups, whilst also being costly, slow, time consuming and requiring highly trained personnel to perform and interpret [1], [80], [81]. Neuroimaging is considered the gold-standard for acute, in-hospital diagnostic techniques and commonplace neuroimaging techniques include computer tomography (CT) imaging or Magnetic Resonance Imaging (MRI) [9]. These are most effective for detecting primary injuries such as skull fracture, contusions, and haemorrhages [75]. Reliance on in-hospital techniques delays diagnosis and therapeutic intervention, risking the patient's neurological recovery. Whilst CT and MRI scans are considered fast procedures lasting les than 60 minutes in most cases, transport-time to emergency departments is unpredictable and waiting times for scans may exceed 4 hours [82].

Neuroimaging is not often performed independently as it can provide false-negative results, for example 29% of TBI patients with negative CT scans show positive MRI findings [83], [84], delaying an already lengthy imaging process. Raised ICP is a common TBI indicator triggered by primary injuries such as intracranial haemorrhages and is a cause of morbidity and mortality after TBI [9], [78]. Normal ICP in adults is 10-15 mmHg, whilst TBI patients may experience raised ICP, with prolonged levels above 20 mmHg [1], [85]. ICP monitoring is routine after initial CT scanning, and is performed by inserting catheters through a cranial access device, or subarachnoid bolt [1], [43]. ICP monitoring can remain in place throughout hospitalisation for initial diagnosis and monitoring to predict outcomes. Patients with severe TBI require neuroimaging to identify injury and planning therapeutic interventions such as ICP monitor insertion, which is an invasive technique.

#### 3) Unmet Need

Current TBI diagnostic pathways frequently result in over-diagnosis and over-triage of TBI, which creates high healthcare costs from the avoidable tests and treatments [86]. In the longer term, over-diagnosis and over-triage of mild and moderate TBI may lead to unnecessary and ineffective treatment causing avoidable side effects and disability status [87]. There is therefore an urgent and unmet need for POC TBI diagnostics, allowing for more informed and specialised management closer to the time of injury in a timely and cost-effective manner. Desirable POC techniques will be non-invasive, avoiding laborious insertion/sample collection and risk of infection commonly associated with ICP monitoring and CSF sampling techniques.

Mild TBI cases are the most difficult to identify and only 5% of TBI cases in U.K. emergency departments each year are moderate or severe injuries based on GCS categorisation [7], which highlights the reluctance to rely completely on GCS triage in the pre-hospital space. This creates an immense but avoidable pressure on emergency departments to triage the moderate and severe cases requiring acute treatment from the mild TBI ones, to improve neurological outcomes and avoid over-investigation and over-treatment.

TBI severity is often variably defined because of injury heterogeneity. Mild TBI can accompany one or multiple symptoms of headaches, nausea and disorientation, lasting from days to weeks [45], and is often overlooked in emergency departments, because of a lack of immediately apparent symptoms. Compounding this, over 90% of patients with mild TBI are never admitted to hospital [72], [88], despite the significant risk of long-term morbidity and the fact that they encompass 90% of all sustained TBI [1], [40], [61], [89].

Many cases of TBI, particularly mild TBI, cause functional and metabolic abnormalities without any detectable structural damage, or damage at a molecular level that is un-detected by neuroimaging [9], [86], [90], [91]. The diagnostic challenges around mild TBI, and consequent lack of appropriate engagement with neuro-rehabilitation services leaves many patients with untreated long-term neurological disorders or disabilities [36], [72], [88], [92], [93].

Moderate and severe TBIs, on the other hand, are accompanied by more obvious and immediate symptoms indicating neurological injury. However, in the context of polytrauma and substance abuse, which may also affect conscious level, this does not guarantee correct identification of TBI or categorisation between mild and severe injuries, especially as both severity levels may coincide with initial unconsciousness [46], eliminating cognitive function assessment. Despite similarities, neurological outcomes after moderate and severe TBI are very different, with in-hospital mortality rates of 10% and 40% [94], [95] respectively. Thus, POC diagnostics need to be not only early, but capable of obtaining information from unresponsive patients and distinguish between moderate and severe cases to allow for intervention and facilities to be prioritised. Of the 30-40% of patients with severe TBI who die, approximately 40% do so within 48 hours of the injury [96], [97] whilst survivors experience neurological disfunction [95], thus early triage and management are key to reducing neurological deterioration and saving lives in this cohort and a quick and accurate diagnostic and monitoring tool could greatly contribute to improving outcomes [98].

Variation in presentation generates different drivers for POC diagnostic techniques. Cognitive techniques fall short for all severities due to mild or delayed effect on mild TBI patients, whilst moderate-to-severe patients experience greater time spans of unconsciousness. Mild cases would benefit from techniques that utilise biochemical responses, as structural changes are less common, whilst early structural changes do not effectively differentiate moderate-to-severe cases, and thus sensitive and specific biochemical responses may aid differentiation. Thus, POC TBI diagnostics would benefit from a sensitive biomarker imaging technique capable of categorising all severity levels. Monitoring the biochemical and metabolic changes over time following initial injury in the acute phase (Fig. 1(bii)), either individually or simultaneously, is where the potential lies for diagnosing and monitoring the presence and severity of TBI. The limitations of imaging biomarkers in detecting these progressive changes (primary and secondary injuries) both in the acute phase and during follow up limit the ability to intervene to improve outcome. The presentation of acute TBI develops over hours after injury, therefore signs and symptoms may exist at different time-points depending on severity. There is also, therefore, an unmet need for a monitoring tool to detect biochemical deterioration both in the acute phase, throughout treatment and during follow up.

### C. The Optic Nerve - ‘the Window to the Brain’

**Fig. 2.**
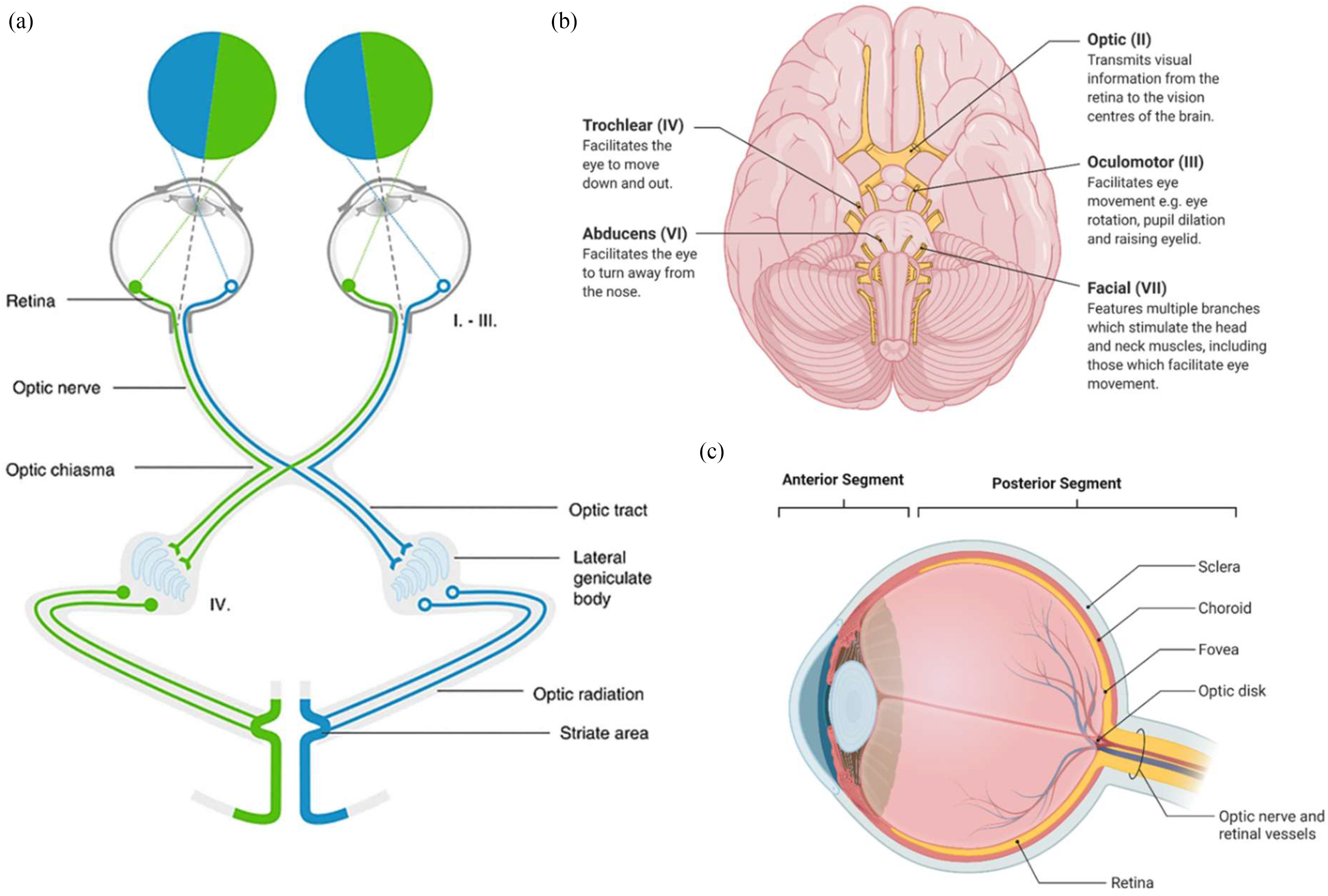
The neuroanatomy of the human visual tract. (a) Photoreceptors in retina transform incident light into changes in membrane potential. This signal is received by bipolar cells and transmitted to retinal ganglion cells whose axons travel to the lateral geniculate body (LGN) in the optic nerve (surrounded with CSF), the optic chiasma and the optic tract. In the lateral geniculate body, part of the midbrain, RGC axons synapse with cells of the optic radiation that travel to the striate cortex (primary visual cortex), reproduced with permission from [101]. (b) Cranial nerves II-VII located within the skull base, responsible for vision and eye and facial movement **Created with BioRender.com.** (c) The posterior segment of the human eye including the choroid with a dense vascular network to supply the outer retina, and the fovea in the centre of the macula containing only cone receptors to allow for sharp images in photopic conditions **Created with BioRender.com**.

The eye, and more specifically the retina, are often referred to as ‘the window to the brain’ [23], [38], [99]. The visual system is linked to the brain by the ON, which consists of axons whose cell bodies lie in the retina, located in the posterior segment of the eye [38], [100]. The neuroanatomy of the human visual pathway is shown in Fig. 2(a), consisting of the retina as well as the ON, optic tract, optic radiation and visual cortex, which are surrounded by cerebrospinal fluid (CSF) [101]. Light enters the eye through the anterior segment and reaches the posterior segment where the retina converts light into electrical signals, which are relayed to the visual cortex through the ON (Fig. 2(c)) [38]. The visual pathway is formed of long axons, vulnerable to stress, and the anterior pathway includes 3 main neuronal types: photoreceptors, interneurons and retinal ganglion cells (RGC). Thirty percent of cerebral cortical neurons are devoted to the visual pathways and visual processing and share multiple functional components with the rest of the central nervous system (CNS) [102]–[104], and thus CNS pathologies are often associated with retinal abnormalities [20]. This has facilitated research surrounding neurodegenerative (ND) disorders and diseases to characterise associated changes in the morphology, movement and chemistry of the eye and visual tract [105]–[107]. This “window” to neurodegeneration in the brain, provides an opportunity for monitoring, triaging scans and diagnoses even before the first symptoms of neurodegenerative (ND) disorders become detectable [20], [108]. The success in this field suggests the potential for the characterisation of other neurological conditions such as traumatic brain injury (TBI), which has a complexity and heterogeneity that makes it difficult for emergency healthcare workers and clinicians to accurately diagnose in a timely manner and thus allocate the correct tailored treatment.

## II. Ocular Responses to TBI and Neurodegeneration

TBI affects the eye either through direct damage to the cranial nerves involved in vision (II) and eye movement, facial muscles and taste (III, IV & VI) (Fig. 2(b)), or dysfunction of the control and regulatory centres of the visual function, which was observed even following mild TBI cases [32], [109]. Head trauma can often result in neuro-ophthalmic deficits though the symptoms can be masked by other secondary injuries [109]. Visual function may be examined clinically in a number of different ways including visual acuity, colour vision, visual field, pupillary function, eye movements and electro-diagnostic testing [110], [111]. Chen *et al.* and others demonstrated delayed progressive optic neuropathy after TBI [112], [113], suggesting that late secondary injury after TBI causes damage to the visual function.

### A. Visual Impairment

Many of the areas of the brain most vulnerable to mild TBI are related to vision [56], including the long axonal fibres connecting the retina to the lateral geniculate body (LGN) and the LGN to the visual cortex, that get distorted by trauma causing diffuse axonal injury (DAI) [47]. There is a further potential for TBI indicators to be found in the retina, which is as an extension of brain tissue with biochemical changes in the retina occurring after various neuronal disturbances [114], and there is potential for ocular manifestations of TBI to be detected by ophthalmic imaging techniques [30], despite the lack of prospective data [115], for example, oculomotor deficits may be, demonstrated by eye tracking in paediatric patients [113], [116]. Fifty four percent of blast induced TBI patients had global visual field loss and visual field dysfunctions were present in all TBI severities [117]. TBI patients described symptoms of photosensitivity, blurred and double vision, decreased visual acuity and visual field defects and patients with depressed visual field sensitivity reported subjectively lower visual quality [117], [118]. In 500 service members with mild TBI there was no difference between the visual dysfunctions and symptoms whether sustained through blast or non-blast mechanisms [56].

### B. Axonal Damage, Blood Brain Barrier Damage, Cerebrospinal Fluid Leaks

After TBI, axonal damage in both the ON and retina is indicated by ON oedema caused by stretching and oxidative stress [119], [120]. Axonal damage is detectable in the ON as early as 1-3 hours following trauma, a possible acute TBI marker, and is present as late as 12 weeks post-injury [119], [120]. Myelin injury in the subacute and chronic phase is evidenced by demyelination of the ON detectable up to 13 weeks after mild, repetitive TBI [118], [121], and in the optic tracts within 7 days, although not in the acute phase [122]. A modified impact acceleration (IA) rat model was utilised to demonstrate blood brain barrier (BBB) damage and axonal swelling an ON segments [123]. BBB damage allows CSF proteins to act as TBI biomarkers in the blood stream and also affects ON tract, worsening axonal injuries developed in the ON after mild TBI [124].

CSF is present in subarachnoid space surrounding the ON and involved in the ocular glymphatic system [101], [125], [126], and may communicate with the eye [127], [128]. Glymphatic dysfunction, characterised by the failure to clear interstitial waste and distribute non-waste compounds in the brain, is linked to neurological pathologies including TBI, mild TBI in particular [125], [126], [129], [130]. Interestingly, Plog *et al.* demonstrated in a murine model that TBI biomarkers may be transported from the brain to blood *via* the CSF in the glymphatic system [131]. Christensen *et al.* explored mild TBI in rats, observing an increase in glymphatic influx and decrease in glymphatic efflux, resulting in CSF being inadequately cleared, contributing to cerebral edema associated with TBI [125].

There has also been a recent increase in research surrounding Spaceflight-Associated Neuro-ocular Syndrome (SANS), which is a series of ophthalmologic and neurologic findings in astronauts following spaceflight. SANS encompasses ON head swelling, globe flattening, choroidal/retina folds and increased CSF volume in ON sheaths [132], [133]. SANS is believed to be caused by elevated ICP and CSF compartmentalisation to the globe or ON [132]–[135], which has been described earlier as a common secondary injury of TBI, thus suggesting similar CSF pathology following brain trauma. SANS has been measured using optical coherence tomography (OCT), MRI, FUNDUS imaging and ultrasound [133], [135].

Killer *et al.* analysed the potential compartmentalisation of the CSF after ON disorders and found that CSF is biochemically continuous up until the ON, but CSF obtained from the subarachnoid space of the ON differed from that surrounding the brain, with both fluids containing different biomarker concentrations [136]. Unfortunately, there is a high chance of contamination in the CSF, adding complications if measuring the chemical response to TBI. Though, CSF leaks are a common complication following all TBI severities, thus the presence alone could be utilised as a TBI indicator [137]–[139], Apkarian *et al.* were able to detect beta-2-transferrin in the subconjunctival fluid with a 25-gauge needle, a marker of CSF leaks [140].

### C. Cell Loss

RGC loss occurs alongside ON degeneration as early as seven days after mild TBI and persist at 6 months post-injury, detectable using OCT both in human and animal studies [123], with the extent of RGC loss being proportional to the TBI severity in animal models, showing the potential of OCT as a diagnostic approach [118]. Axonal injury within the ON may or may not drive RGC loss, which may also be affected by cerebral primary and secondary injury [141].

Many systemic and cerebral neurodegenerative (ND) diseases manifest in the eye including Alzheimer's disease (AD), a form of dementia most commonly associated with memory disturbance, motor neuron disease (leading to muscle weakness), Parkinson's disease (PD), another form of dementia presenting as a movement disorder and Multiple Sclerosis (MS), a CNS disorder that causes vision, motor and sensory difficulties [20], [38], [106], [142].

Structural and physical ocular changes may be measured in the “early” phases of neurodegeneration [143]–[146]. For instance, Cheung *et al.* linked clinical retinal diseases with dementia, including macular degeneration, glaucoma, and diabetic retinopathy, which develop from biochemical pathologies that could be identified even before cognitive decline and changes in vision become apparent [21], [100], [103].

Eye movement and afferent visual abnormalities are amongst the earliest clinical manifestations of ND diseases, including PD [38], [143], [147], AD [24], [104], [105], [147] and MS [106], [148]. Eye-tracking techniques are typically used to identify oculomotor impairments [147], with strong evidence that eye movement disorders associated with the neurodegeneration could provide diagnostic information or disease progression evaluation in PD and MS [106], [149]. In addition, eyelid function is also abnormal in some ND disorders, being present in patients with neurodegeneration such as PD, Huntington's Disease, Progressive Supranuclear Palsy (PSP) and Chronic Progressive External Opthalmopledia identifying disturbances to blinking rates, eyelid retraction and ptosis *(*droopy eyelid) [143].

### D. Optical Coherence Tomography Detectable Changes

The relationship of changes in ocular structures, such as the retina, ON, choroid, pupil, and lens as well as tears, with neurodegenerative diseases has been extensively reviewed elsewhere [20], [21], [23], [24], [148], [150], [151]. TBI, AD, MS, cerebrovascular disease, and PD patients suffer retinal nerve fibre layer (RNFL) and ganglion cell layer thinning (indicating RGC loss), reduced retinal vasculature, and increased neuronal plaques in the visual pathways [20], [23], [89], [103], [105], [152]–[154]. Such changes are typically monitored through OCT, an *in-vivo* ocular imaging technique that utilises laser interferometry [38], [103], [105], [148], [154].

The retina provides a convenient window to assess neuronal and vascular changes in the CNS [21], [24], [100], [103], [108], [154]. Frost *et al.* suggested that retinal amyloid-Beta tests can differentiate between the presence of AD with 100% sensitivity [107], and this association between ND diseases and early biomarkers including the amyloid-Beta, tau proteins and inflammation has been further echoed in more recent reviews [21], [22], [24], [25], [104]. Further cellular changes investigated alongside the ND disorders have been the microglia activation leading to neuroinflammation, which translates into retinal inflammation [100], [108].

The retina and ON (Fig. 2) can undergo structural changes, unrelated to mechanical shear [155], but correlated with the loss of post-synaptic neurones having detrimental effect on RGC, a process termed trans-synaptic degeneration and resulting in retinal thinning [115], [156].

### E. Retinal Thinning and Changes in Blood Vessel Morphology

**Fig. 3.**
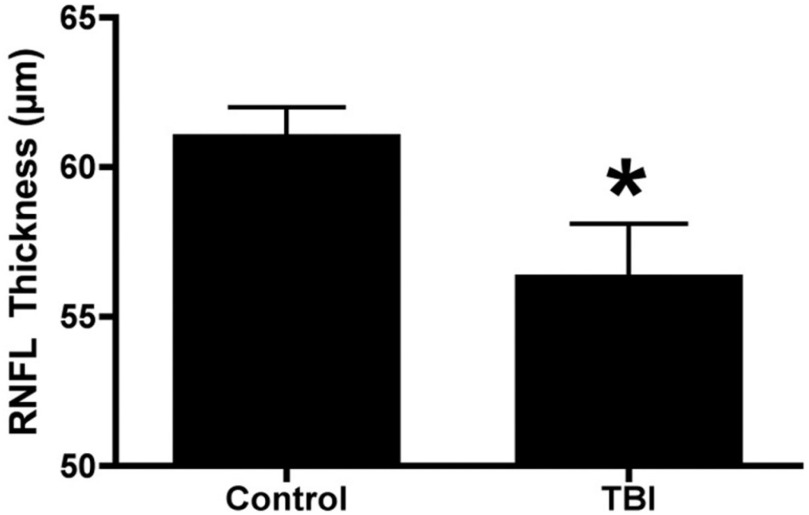
OCT measurements of control and indirect, blast mediated TBI, murine eye samples, illustrating significant loss in the peripapillary RNFL thickness, reproduced with permission from [120].

CT scans and ultrasound have identified ON sheath distention in all TBI severities, related to elevated ICP [157]–[159], which may also be associated with papilloedema (swelling of the optic disc). Even athletes who partake in high-impact sports with no history of even mild TBI, show signs of ON damage [160], suggesting that it would be difficult to standardise TBI diagnostics without symptoms to warrant scans. Multiple studies demonstrated either the RNFL thinning or thickening using OCT after TBI [113], [120], [161]–[163], with Fig. 3 illustrating significant loss in the peripapillary RNFL thickness [120], as well as reduced sub-foveal choroidal thinning after mild TBI, which also associates with disease severity in other ND disorders [105], [163]. However, these structures are also known to be affected by age and therefore age-adjusted normative databases are required. Retinal photography has also detected changes in the morphology of retinal vessels and fractal analysis (markers of cerebral vascular changes post mild TBI), with increased arterial and venous tortuosity in the acute period and increases retinal venular calibre [114], [164].

### F. Inflammation - Histology, Computerized Tomography and Magnetic Resonance Imaging

An inflammatory response within the brain can affect the retina *via* the ON [100]. After TBI, microglial activation (indicator of neuron damage and poor CNS health [165]) in both the optic tract and the retina, indicated inflammation, within the subacute period (2-7 day) after TBI and inflammation is detectable up to 2 weeks following trauma [111], [123], [166]. In a histological study of murine eyes and brain subjected to TBI and ocular blast injury, neuron loss biomarkers were not identified, and neuroimaging (CT and MRI) was normal, however although inflammation was present after both eye and brain injury [122], [166]. Thus, neuroinflammation of the retina and ON may evolve as a diagnostic measure of the pathological changes in the brain following TBI as the states of the two are closely related.

Whilst most studies focus on structural changes, biochemical responses will develop much more rapidly than structural changes and may be detectable much earlier in the ND disorder pathogenesis [99], suggesting that TBI indicative early-stage biochemical biomarkers being detected, identified and characterised in the ocular tract.

## III. Chemical Responses to TBI

Identifying chemical responses to TBI and correlation with injury severity would enable a better mechanistic understanding, development of new diagnostic modalities and opportunities for more effective therapies. Recent publications investigate chemical responses to TBI for early and accurate diagnostics [40], [66], [167], [168], and the capability to detect changes as early as an hour after trauma [169]–[172], in biofluids such as blood, CSF, urine and saliva [173]–[177], using molecular sensing techniques [178]. Changes present in biofluids can be termed as biomarkers and concentrations correspond to changes in metabolism, vascular function, inflammation, extracellular matrix status and damage to axons, neurons and glial cells after injury [80], [179], [180]. TBI is heterogeneous without symptoms that define injury severity levels, however, even mild TBI can produce short periods of neurological dysfunction [173], which can be detected using biochemical measurement techniques. Since mild TBI is the most frequent injury severity sustained [61], [89], reliable biomarkers at early stages post-trauma are essential, and increasing evidence indicates that the biochemical biomarkers may be both sensitive and correlate well with injury severity [74].

NICE guidelines recommend focussing research on biomarkers for diagnosing, monitoring progression and stratifying patients with TBI for therapies [7]. Acute, mild to moderate TBI biomarkers in blood have been validated with high specificity to negative neuroimaging with the potential to eliminate 39% of unnecessary scans [83], [181], potentially saving 39-71 Euros per patient [82]. Biomarker-based triaging could focus subsequent hospital-based neuroimaging on those patients at greatest need, avoiding unnecessary radiation exposure and facilitating significant savings to the healthcare providers [83], [182], [183]. Implementing accurate diagnoses through TBI biomarkers in the acute phase and, especially, at the POC could allow better, faster, and more efficient management through more accurate and appropriate triage, decision-making, and management [174], [184].

### A. Biomarkers for TBI Diagnostics

Many TBI biomarkers are assayed in accessible biofluids [40], [66], [80], [167], [173], [175], [183], [185]–[187]. Pineda *et al.*, stated that ‘good’ biomarkers should be in easily accessible biofluids, with low background levels in healthy control groups, have high sensitivity and specificity to all injury severities and be released in a “time-locked sequence” after the injury [40], [188], [189]. In addition, the response time, clearance, and half-life of a biomarker must be considered, all of which give indication of the timeframe during which it can be detected post injury and the expected final baseline concentration in bodily fluids [175]. The most commonly used biofluids for TBI biomarker detection are blood and CSF, which contain molecular patterns representative of the CNS [190]. Blood samples are most accessible in the acute, POC settings although CSF samples may better model the brain's response to TBI being on the brain side of the BBB [190], however this becomes permeable almost immediately after even very mild trauma [124]. Nevertheless, biomarker concentrations in CSF show significant interpatient variability [191], making diagnostic procedures difficult to standardise.

Although well recognised and validated, acute TBI biomarkers may overlap with other disease and injury processes, such as extracranial plasma or serum, proteins released from damaged cells, polytraumatic organ and muscle injury [66], [192], lung tumours [193], and subclinical ND conditions [74], [109]. Despite this however, in 2007 the Scandinavian healthcare system introduced S100B as the first brain biomarker to be used within clinical practice guidelines to predict negative CT scans, aiming to reduce the number of unnecessary scans on patients without significant TBI [82], [187], [194].

**Fig. 4.**
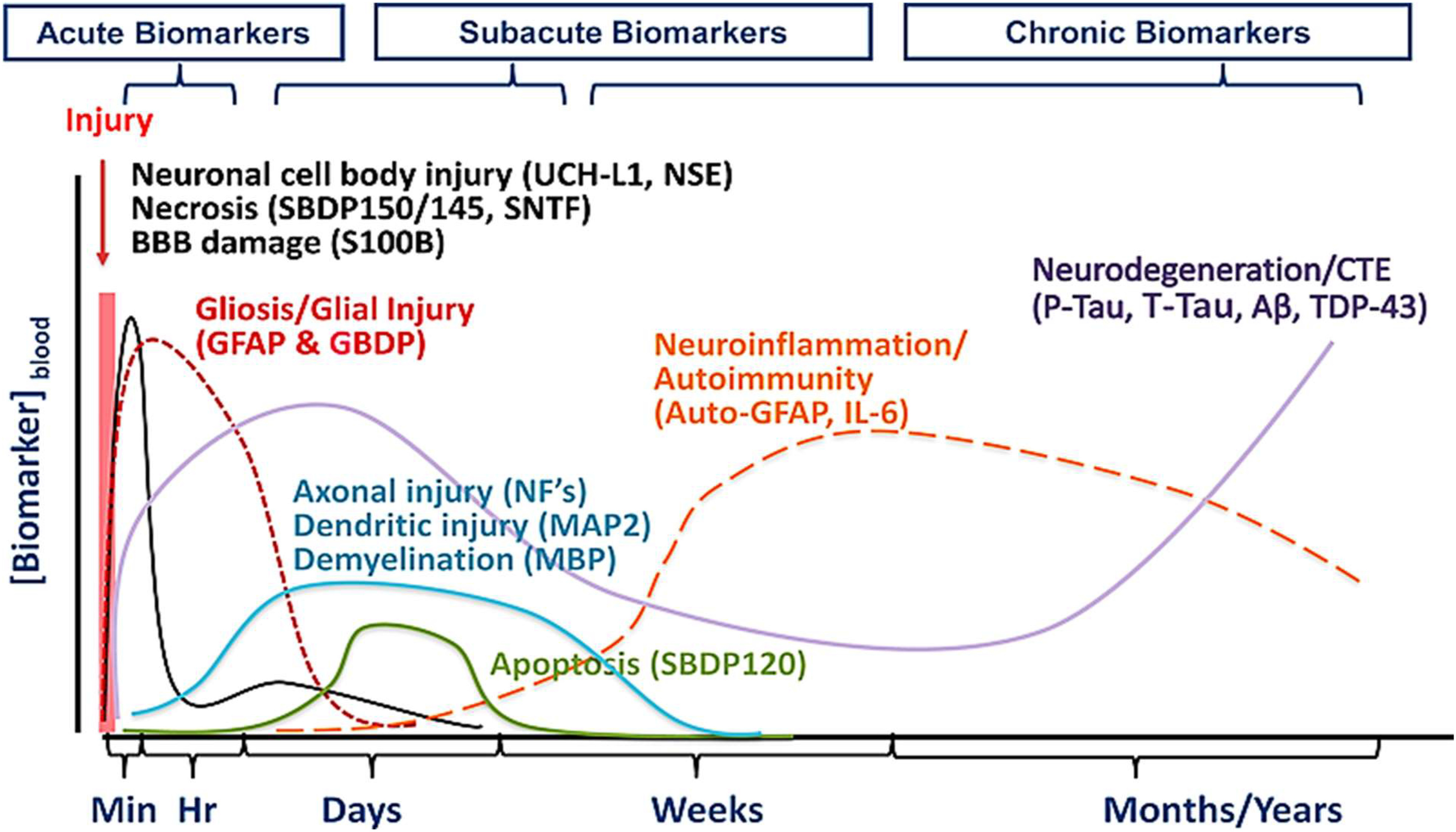
Illustration of the concentrations of validated TBI biomarkers in the acute, subacute, and chronic phases following the primary injury. Early, on-site diagnostics would be completed within minutes to hours following injury and thus, necessitates the need to be sensitive to acute-phase indicative biomarkers, reproduced with permission from [40].

Recognised biomarkers measured in the acute phase after TBI include, S100B, glial fibrillary acidic protein (GFAP), neuron-specific enolase (NSE), Ubiquitin C-terminal hydrolase-L1 (UCHL1), total tau (t-tau) protein and αII-spectrin breakdown products [40], [173], which align with standard TBI diagnostics, for example measuring raised concentrations of GFAP, S100B, UCHL1 NSE and t-tau in blood when abnormalities are present in CT scans [174]. Fig. 4 summarises the biomarkers identified with TBI progression, categorising them into acute, subacute and chronic phases after TBI [40]. Table I outlines these biomarkers along with some of which are emerging in the field of TBI, categorising the associated location, biofluid, sensitivity, specificity and injury severity for each.

**TABLE I:** Outline of the Recognised and Emerging Biomarkers of TBI With Their Respective Locations, Sensitivities, Specificities and Associated Injury severities. GFAP = Glial Fibrillary Acidic Protein; UCHL1 = Ubiquitin C-Terminal Hydrolase L1; NSE = Neuron-Specific Enolase; T-Tau = Total tau; NAA = N-acetyl-aspartate; GSH = Glutathione. Sensitivity/Specificity: High = ≥85%, Moderate = 50-84%, Low = <50%

**Biomarker**	**Origin**	**Biofluid**	**Timeframe**	**Sensitivity**	**Specificity**	**Severity**	**References**
S100B	Calcium-binding protein expressed in glial cells	Blood, CSF	Acute	High	Low	Moderate, Severe	[61], [74], [429], [173], [175], [183], [189], [191], [195], [196], [200]
GFAP	Structural filament protein of astrocytes	Blood, CSF	Acute	High	High	All	[40], [61], [196], [203], [429], [430], [74], [169], [173], [175], [183], [189], [191], [195]
UCHL1	Cytoplasmic enzyme expressed in neurons	Blood, CSF	Acute	High – less sensitive to mild TBI	Moderate – not CNS specific	All	[40], [61], [429], [431], [432], [66], [74], [173], [181], [185], [210], [211], [226]
NSE	Glycolytic enzyme expressed in red blood cells	Blood, CSF	Acute	Moderate – limited evidence for mTBI	High – to the brain	All	[40], [61], [169], [173], [185], [208], [429], [433]
αll-spectrin	Cytoskeleton protein found in axons and presynaptic terminals	CSF	Acute - Chronic	Moderate – limited evidence for mild TBI	Low	All	[40], [173], [188], [212], [248], [434]
t-tau	Microtubule-associated protein expressed in neurons and astrocytes	CSF	Acute - Chronic	High	High	All	[74], [83], [173], [174], [203], [216], [217], [435]
NAA	Amino acid derivative located in neurons	Blood, CSF	Acute	High	Moderate	All	[49], [231], [234]–[238], [436]
GSH	Non-enzymatic antioxidant present in cells	Blood, CSF	Acute	High	Moderate	All	[244]–[247], [251]
MicroRNA	Small molecules regulating gene expression	Blood, CSF	Acute - Subacute	High	High	All	[184], [189], [218]–[224], [437]

The most widely investigated acute biomarkers include S100Β, a calcium-binding protein expressed in glial cells of the CNS regulating calcium level, and GFAP, a structural, filament protein of astrocytes located in the CNS CSF, associated with astrocyte and BBB injury [61], [173], [175], [183], [189], [191], [195], [196]. Acute phase S100B is a powerful TBI biomarker because its concentrations are elevated in CSF and serum, by astroglial injury [173], and align with CT abnormalities [40], [182], [197], [198], as well as raised ICP and poor outcomes [195], [199], [200].

As S100B is widely researched and documented, the healthy baseline concentration in adult serum is stated and agreed to be approximately 0.11 ug/L [178], [198], [201]. However, the literature surrounding other sample types and TBI severity are varied. Studies tends to focus on elevations and subsequent fluctuations in biomarker concentrations following injury, making each one context specific. Even in the case of three studies, all measuring S100B serum concentrations after mild TBI within the acute phase using immunoassays; all conclude mild levels to be 0.31 ug/L, 0.043 ug/L and 0.39 pg/mL [84], [198], [202]. These discrepancies are worsened by different sample types, timeframes, storage methods, patient age and measurement techniques, and in-depth discussions surrounding each TBI biomarker behaviour post-injury are beyond the scope of this review article. These inconsistencies have led to confusion in characterising the biochemical response, which is detrimental to refinement of new techniques. This highlights the value of a technique that utilises repeated, time-sensitive measurements or of biomarker panels that allow concentrations to be assayed as ratios to one another.

GFAP is brain-specific and raised concentrations in the CSF [40], [169], [173], [203], [204], appear as early as 1-hour post-injury [169]. GFAP is the most discriminatory acute, TBI biomarker for predicting CT abnormalities [174], [182], [197], [205], also associating with inflammation [206], and patients who have sustained blast related TBI [207], having been cleared by the US Food and Drug Administration (FDA) to predict the need for CT scans within 12 hours of mild TBI [197].

Biomarkers associated with neuronal damage include NSE, a glycolytic enzyme and UCHL1, a protease expressed in red blood cells (RBCs) and CSF, respectively [40], [61], [173]. NSE is correlated to posttraumatic inflammation [185], and raised NSE levels, within 4 hours of moderate-severe TBI may identify patients at higher risk of cognitive dysfunction [208]. The detectability of NSE in RBC makes it readily accessible but introduces a risk of cross-contamination in blood samples in the event of haemolysis (RBC rupture), rendering it less specific [209].

**Fig. 5.**
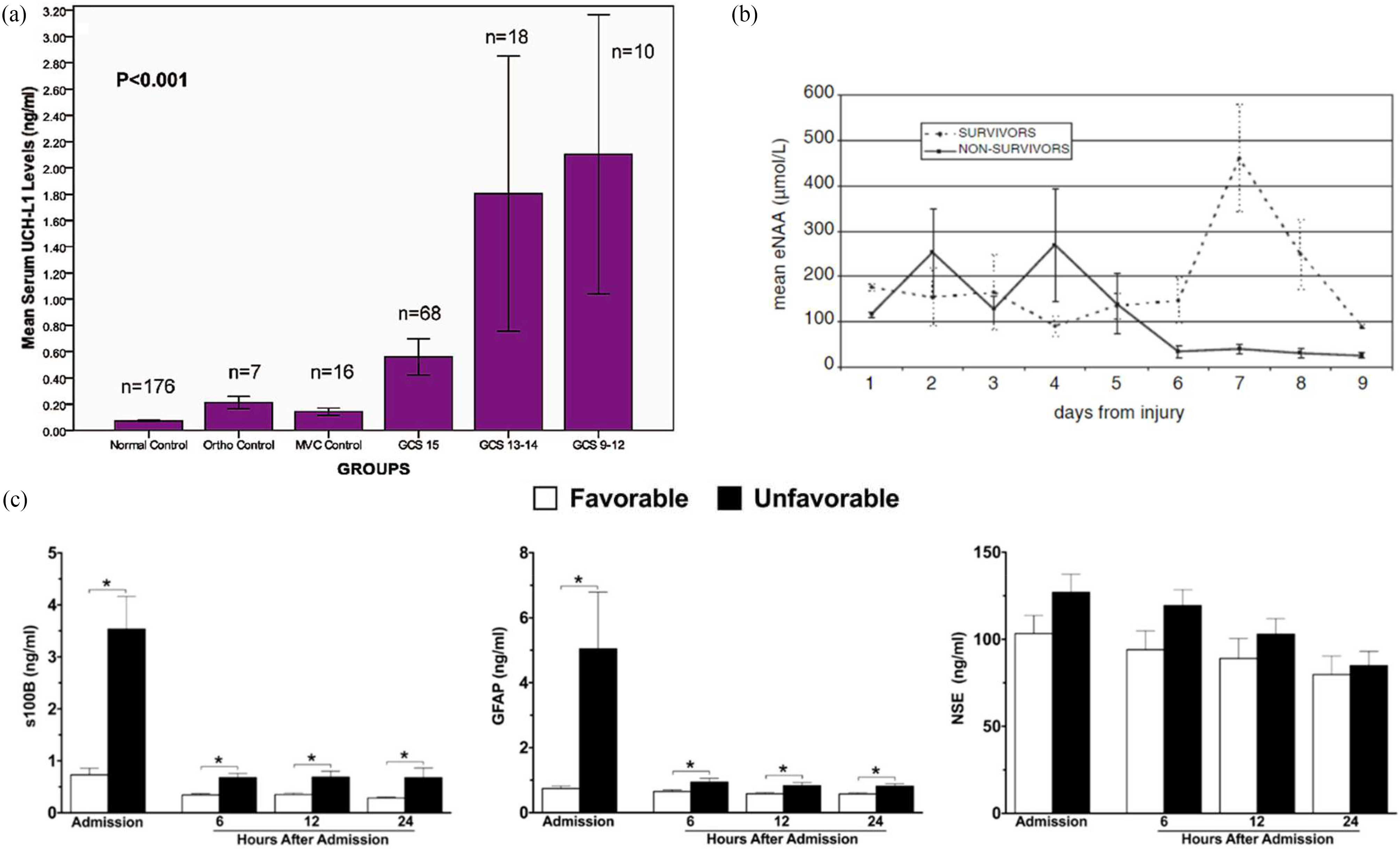
(a) Mean UCH-L1 levels in uninjured and in TBI patient groups, illustrating increased levels after trauma, with measurements taken from serum 4h post trauma. Analysed using an enzyme-linked immunosorbent assay (ELISA) kits, reproduced with permission from [170]. (b) Time-course of eNAA using microdialysis in severe TBI survivors and non-survivors, demonstrating irregular levels in non-survivors and low levels followed by a large spike in survivors [235]. (c) Plasma concentrations of S100-B, GFAP and NSE of moderate-severe TBI patients within the first 24 hours of hospital admission [199].

UCHL1 is a cytoplasmic enzyme found in a high abundance in neurons [66], [170], [172]. Studies have identified elevated levels in plasma after mild to moderate injuries (Fig. 5(a)), as early as 1-hour post-injury, associated with axonal damage and DAI [170], [172]. UCHL1 is sensitive to patients with positive CT scans and unfavourable outcomes [181], [185], [210], but has low specificity, in the CSF, blood, peripheral nervous system, limiting its utility [66], [211].

*αII*-spectrin is a cytoskeleton protein in axons and presynaptic terminals [212]. Calcium activated products formed during *αII*-spectrin protein degradation in response to necrosis (uncontrolled cell death) are a potential TBI biomarker for secondary injuries such as, ischaemia [40], [173], [175], [213].

Total tau (*t-tau*) protein is a microtubule-associated protein expressed in neurons and astrocytes [173], and associated with ND disorders including AD [214], [215]. *t-tau* levels are elevated at various injury severities [174], [203], and in association with DAI [83], [216], as early as 1 hour after TBI and correlated with positive findings on CT [185], [217], as well as self-reported symptoms after mild TBI [66].

Micro RNA (miRNAs) are small molecules that contribute to gene expression regulation, blood, CSF and brain tissue levels [184], [189], [218]–[222]. It has been demonstrated that miRNA expression is altered across all TBI severities over time periods ranging from 1 hour to several days post-trauma [184], [223], [224].

Plasma S100B and GFAP remained elevated throughout the entire 24-hour period after TBI and were consistently more elevated in TBI patients with unfavourable outcomes (Fig. 5(c)) [199]. Both astroglial injury biomarkers combined have the potential to be used as a duo-marker panel for reliable and sensitive TBI diagnostics and outcome predictions, or with other multiple biomarkers such as UCHL1 and GFAP to give high diagnostic accuracy [225]–[229] Nevertheless, using biomarkers in tandem does not always outperform S100B or GFAP alone [174], [199]. On the other hand, UCHL1 may be the dominant blood biomarker, with little additive effect of combining with GFAP and S100B [181]. Ongoing studies towards the clinical application of these biomarkers for TBI diagnostics [230], combined with the advancements in biomarker sensing techniques and increasing data on the biofluid markers correlating with underpinning pathologies, suggest a great promise for future TBI diagnostics.

### B. Emerging TBI Biomarkers

N-Acetyl-Aspartate (NAA) is a highly abundant amino acid derivative in adult brain neurons [231], [232], produced by neuronal mitochondria and thus, levels reflect mitochondrial oxidative metabolic status and stress [233]–[235], and is therefore a biomarker of neuronal and axonal function and loss [49], [235]–[238]. DAI, direct injury and ischemia are a frequent cause of neuronal injury [49]. Shannon *et al.* utilised microdialysis to establish elevated extracellular NAA levels after TBI due to an efflux of NAA due to adverse brain chemistry which then decreases in the 50-96 hour period post-injury [231]. This fluctuation in eNAA levels over the subacute time-course of a TBI was explored by Belli *et al.*, and illustrated in Fig. 5(b), reporting low-levels in severe survivors followed by a large peak, whereas non-survivors expressed irregular levels throughout [235]. Fu *et al.* demonstrated decreased NAA concentration in normal-appearing white matter after axonal damage, linking NAA levels with mitochondrial dysfunction and acute inflammatory lesions [233], [239]. Malisza *et al.* found that in ischaemic rat brains, there was a continuous decrease in NAA levels in animals with tissue death [240], replicated in other TBI studies [231], [237], [241], [242], with brief elevation after insertion of a cerebral catheter for microdialysis and polyethylene catheters for blood sampling and pressure monitoring [231], [232], [243]. NAA is also detectable at POC using a non-labelled lab-on-chip device and Raman spectroscopy to measure an immediate NAA spike in severe TBI patient blood samples up to five control group levels, decreasing over 24 hrs post-injury [234].

The BBB is compromised in the acute phase after head injury with increased glutathione (GSH) levels [244]. GSH is a non-enzymatic antioxidant present in cells, that has a role in protecting cell membranes from oxidative damage and maintaining the integrity and function of the BBB [245]–[248]. Low GSH suggests oxidative damage and can initiate oxidative stress-mediated neuronal loss, and associates with neurological disorders including AD, PD, MS and TBI [247], [249], [250]outlining GSH as a potential acute TBI biomarker, although variable GSH levels in healthy control groups and potential of artifacts in detection may limit utility [251].

### C. Potential TBI Biomarkers Associated With the Eye

Levels of GFAP and UCHL1 increased in plasma after direct trauma in a porcine ON crush model [252]. Table II summarises ocular changes associated with TBI and the pathologies that alter indicative biomarkers that may be present in ocular structures, blood or CSF after trauma.

**TABLE II:** Potential Biochemical Ocular TBI biomarkers. The Strongest Biomarker Candidates Have Been Included Because of Their Specificity and Sensitivity to TBI Severity

**Ocular Manifestations of TBI**	**Strongest Biomarker Candidates**	**References**	**Associated Biomarkers**	**References**
**Neuronal and Axonal Damage**	t-tau, NAA	[47], [83], [119], [121], [173], [235]	αll-spectrin, GSH, NSE, UCHL1	[40], [47], [61], [66], [119], [121], [171], [212], [249]
**Retinal Ganglion Loss and RNFL thinning**	t-tau	[123], [438]–[440]	NHS, GSH, NAA	[123], [441]
**Neuroinflammation**	NAA (axonal swelling), S100B (microglial cell activation)	[111], [233], [442]–[444]	NSE, GFAP	[185], [206]
**Chemical Response in CSF**	t-tau, GFAP, NSE	[40], [173], [196], [203], [438]	UCHL1, S100B, αll-spectrin, miRNAs	[40], [61], [188], [189], [196]

*t-tau* and NAA are potential candidates, since *t-tau* has been identified in the retina in AD [21], [22], [24], [25], [104], and NAA is sensitive to ocular pathologies after TBI such as neuronal and axonal damage [236], [237]. *t-tau,* NAA, *αII*-spectrin, GSH and NSE are all expressed in or associated with neurons and axons [83], [173], [212], [249], and their release after TBI therefore suggests neuronal and axonal damage [121], [235], such as retinal ganglion loss and RNFL thinning [119], [123], [161]–[163]. NAA, NSE and GFAP are also associated with inflammation [185], [206], [233], which is also an ocular response to TBI, particularly within the ON [111], [123], [166].

Disproving CSF compartmentalisation would suggest the possibility of biomarker detection in the CSF such as, the GFAP [40], [173], [203], UCHL1 [40], [61], S100B [61], [196], αII-spectrin [188], and MicroRNAs [189], in the retrolaminar CSF (behind the ON head) and potentially in the eye. This is particularly applicable to the detection of GFAP, since it is a marker of BBB dysfunction [40], [173], [203], which facilitates the CSF leakage [124].

## IV. Biomarker Detection Techniques

Developments in correlating TBI biomarker levels with trauma severities would constitute a major step towards more accurate identification and understanding of head injuries, particularly mild TBI or concussion [188]. This would also enable a better grasp of the underlying molecular mechanisms and signalling pathways, facilitating improvement in management, recovery and potential drug treatments [188], [253]. Biomarker detection methods discussed below have the potential to be implemented for continuous monitoring throughout the various stages of TBI, unlike the aforementioned common TBI diagnostic methods, many of which are invasive, time-consuming, expensive or potentially harmful due to the ionising radiation or risk of infection. Though biomarker readings can be seen as single time-point measurements, work is ongoing to develop kinetic models of TBI biomarkers to predict the concentration trajectory up to 12 hours following injury [178].

These molecular sensing techniques all exhibit attributes as potential routes for biomarker detection and TBI diagnostics however currently, no single method has the capacity to measure *in-vivo* biofluids rapidly, effectively, and non-invasively. Urine is a potential *ex-vivo* biofluid for detection of brain biomarkers [176], however, it has low specificity, varying sample volumes and indirect route for sampling, making it often highly diluted once released from the kidney and requiring patient's cooperation [169]. Saliva has also been investigated as a potential biofluid for TBI biomarkers [173], [177], and whilst it is easy to collect non-invasively, only S100B and UCHL1 have been so far successfully measured from it [167], with the analysis typically including an additional step of exosomes isolation thus, introducing additional challenges for real-time measurements and diagnostics [254]. Biomarker sensing techniques which require *ex-vivo* sampling incorporate additional risk of samples being affected by preparation and preservation methods as was illustrated by Abdelhak *et al.* who has freeze- thawed CSF samples over 5 cycles showing a 50% decrease of the GFAP within those [255].

Recently, the FDA approved the Banyan Trauma Indicator (BTI), a novel biomarker screen to aid triaging of mild to moderate TBI patients before undergoing CT scans, with the intent of reducing costs and exposure to radiation, along with increasing availability to other patients. The BTI measures the UCHL1 and GFAP levels in blood samples, indicating the presence of intracranial lesions in the form of positive or negative assay results [256], [257]. A study was carried out to determine the cost-effectiveness of this product, concluding that for moderate injuries, the test would need to considerably cheaper [256], [258]. The test utilises limited biomarkers that have been detected within an hour of brain trauma and reportedly it takes 3-4 hours for results to become available [256], [258].

**Fig. 6.**
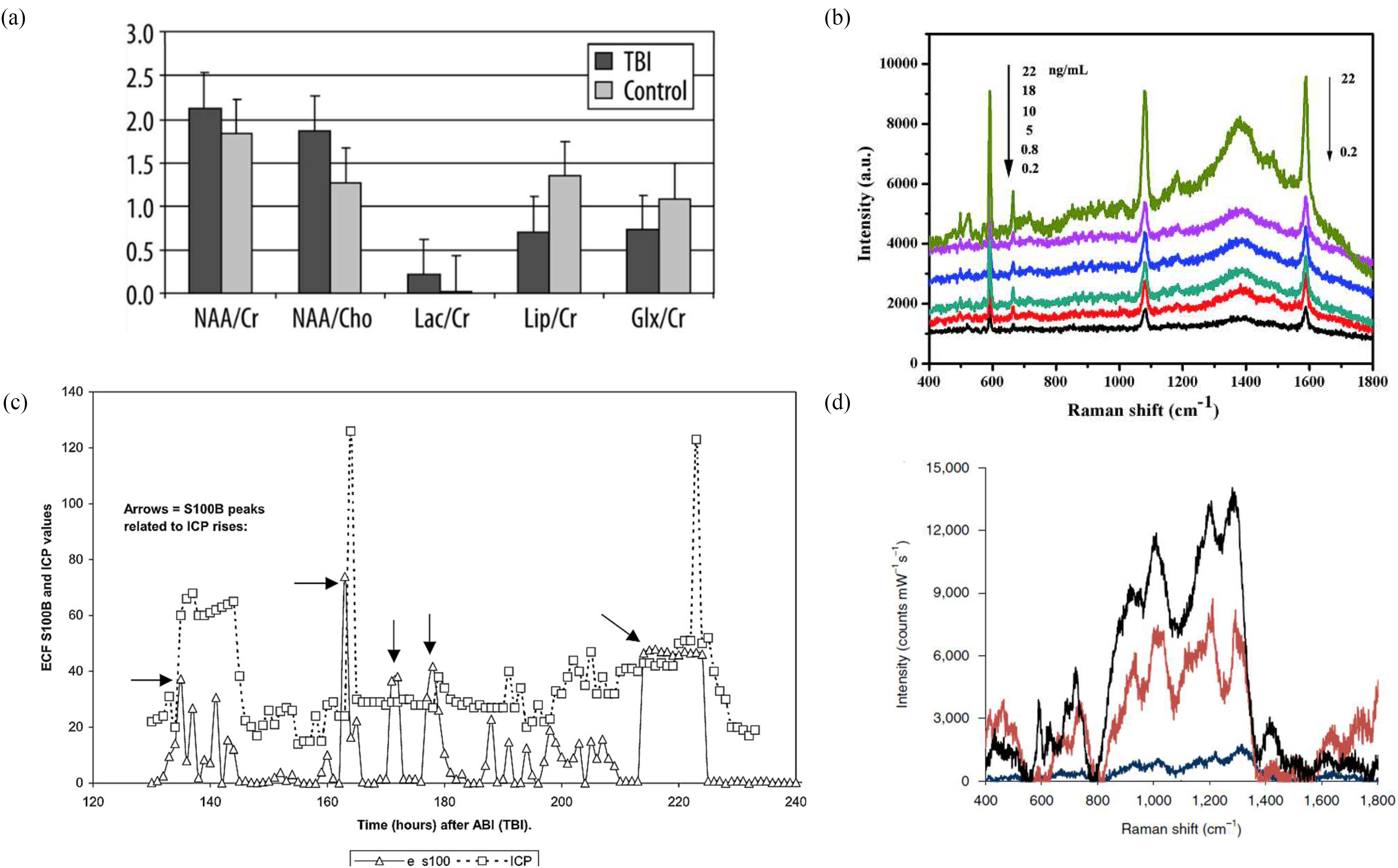
Examples of chemical sensing techniques used to measure TBI biomarkers. (a) NAA levels measured as metabolite ratios in mild TBI patients and healthy volunteers using proton MRS. Measurements were taken 1-20 days following trauma and indicate higher NAA concentrations in mild TBI patients than controls [261]. (b) Surface Enhanced Raman Spectroscopy (SERS) used to measure ex-vivo samples, determined that SERS can detect a clear change in spectra when measuring multiplex immunosensors after incubation with varying concentrations of S100B and NSE [168]. (c) In-vivo measurements of S100B, from brain extracellular fluid, using MD. S100-B levels peak in alignment with periods of raised ICP following TBI, reproduced with permission from [195]. (d) SERS used to measure NAA concentrations in finger-prick blood plasma samples at t = 0 (black) and t = 8 hrs (red) following TBI, compared to healthy volunteers (navy) [234].

In clinical settings, optical techniques are favoured for being highly sensitive, non-destructive and rapid. Optical brain imaging methods capable of identifying microscopic structure and function have been continuously developed over the past 40 years since Jöbsis first measured brain blood and tissue oxygenation using near-infrared light [259], [260]. We summarise some of these techniques below as they show potential for translation to the ocular system, and Fig. 6 overviews how some of these techniques have been used in literature to detect or monitor TBI biomarkers to date [261].

### A. Mass Spectrometry

Mass spectrometry measures the mass-to-charge ratio of gas-phase ions, which involves ionising the sample to break down molecules into fragments of separate masses and analyse them [190], [262]. However, this method typically requires samples to be analysed under a vacuum and to be ionised, limiting its use in continuous, *in-vivo* measurements due to difficulties in maintaining physiological states of biological samples [262]; as continuous measurements are necessary for determining TBI severity, this limits the potential of mass spectrometry as a TBI monitoring technique.

Mass spectrometry is often compared to another metabolomic technique, nuclear magnetic resonance (NMR), which can be utilised *in-vivo* when combined with magnetic resonance spectroscopy and capable of non-destructive sample analysis, but has a lower sensitivity than mass spectrometry [263]. Such comparisons highlight the strengths of mass spectrometry, including the large number of metabolites its capable of detecting and low skill required to implement it (ideal for POC settings). However, of the requirement for extensive sample preparation and sample destruction prevent *in-vivo* applications and greatly limit use pre-hospital or to obtain real-time monitoring data [264]. Mass spectrometry can be applied to microdialysis samples (MD) which is obtained by a catheter inserted through a lumen into the brain, where perfusion fluid is pumped through the catheter [195]. The tip of the catheter allows diffusion of sample fluid along a concentration gradient to equilibrate with the perfusion fluid, mimicking a blood vessel and sampling extracellular fluid without blood withdrawal [265]. The extracted fluid may also undergo high-performance liquid chromatography, resulting in separation of the chemical components [231], [266]. Studies have successfully detected the indicative TBI biomarkers using MD including for instance, S100B and NAA [231], [235], [243], [245]. However, artefactual disruption from catheter insertion with increase in injury markers has been observed in many studies [231], [232], [242], [243], pointing towards an inconvenient waiting period after catheter insertion to allow biomarkers to reach a steady state.

### B. Magnetic Resonance Spectroscopy

Magnetic resonance spectroscopy (MRS) is an *in-vivo* analytical technique that non-invasively interrogates brain tissue metabolism. Similar to MRI, radio-frequency electromagnetic radiation is used to excite nuclei into alignment and then stopped to allow nuclei to return to their original state, creating a fluctuating magnetic field which is detectable as a current induced in the receiver coil [238], [267]. In a complex chemical environment, nuclei are shielded from the main (external) magnetic field by the electrons surrounding the nucleus. This electron shielding results in small changes of the frequency of the MR signal detected and is called the chemical shift and is the basis for metabolite identification using MRS. This creates an output in the form of a spectrum which provides the information on which molecular bonds are present in the sample in the form of spectral bands located at unique wavenumbers, which are split into regions representing vibrational modes and with intensities indicating the relative concentration of each molecule [268].

Proton MRS uses the radio-frequency signal from MRI to obtain a chemical shift from a sample, which indicates its metabolite concentration *via* spectral peaks [75], [238], [269], obtaining the biochemical information of an otherwise morphological scan [237]. Were proton MRS pursued for TBI diagnostics then it could be easily implemented within a pre-existing MRI facility, which are available in most western hospitals. Proton MRS is the most used MRS technique for studying brain metabolism after TBI and measures several metabolites. Several targets of proton MRS may have value as prognostic indicators including NAA, choline, myoinositol and lactate [237], [270]–[273]. Of these NAA and choline are decreased and increased in severe injury respectively, and together may be the most reliable indicators of eventual outcome [270].

MRS holds great potential in the post-injury follow up period, when it may be useful for monitoring neurodegeneration, which can complicate the long term follow up of patients with TBI [274]. However, there are drawbacks of MRS that may deter researchers from establishing standardisation. Patients with restlessness preventing them from lying still for a prolonged time are excluded or must undergo anaesthesia to ensure they are stationary, delaying diagnoses and potentially interfering with other assessments [237], [238]. Proton MRS also requires specialised knowledge to align and calibrate each measurement, along with a well-trained, dedicated personnel to regularly service it.

### C. Fluorescence to Detect TBI Biomarkers

Fluorescence occurs when incident light undergoes the Stokes Shift, in which the scattered light has longer wavelengths than that of the source [275]. Whole-body fluorescent imaging has been performed on cryolesioned mouse TBI models to measure apoptosis using near-infrared molecular probes and tracers [276]. Fluorescent microscopy can also be used to monitor apoptosis (cell death) after TBI and has been done so to image Rabbit anti-AIF and anti-EndoG antibodies [277], [278]. Inverted fluorescence microscopy has been used in animal studies to image nitrogen monoxide (NO) in real-time [279], BBB degradation increases NO levels, an important TBI free radical, however, there are currently few viable techniques for *in-vivo* imaging of NO brain concentrations in humans [280], though there has been success in ophthalmic applications.

Cordiero *et al.* have utilised fluorescent signals with confocal laser scanning ophthalmoscopy to single retinal nerve cell apoptosis *in-vivo* and termed the technique detection of apoptosing retinal cells (DARC) [281]–[284]. DARC was developed to diagnose glaucoma in the early stages before vision loss and has been translated into humans to identify retinal cell apoptosis in retinal images of glaucoma patients, finding DARC counts were significantly higher (correlating to a greater number of apoptosis) in glaucoma patients compared to healthy controls, and even more so for those with increased disease progression, indicated by the optic disc, RNFL and visual parameters [285]. Fluorescent microscopy combined with ophthalmoscopy has the potential for monitoring DARC counts *in-vivo* in the acute phase of TBI to diagnose and characterise injury severity, but require numerous patient preparation steps, firstly being a single injection dose of ANX776 to visualise cells during imaging, followed by pupillary dilation.

Bermond *et al.* imaged human retinal pigment epithelium (RPE) cells from *ex-vivo* fovea, perifovea and near-periphery, using confocal fluorescence microscopy [286]. Whilst Fundus Autofluorescence (FAF) is proving a promising tool for monitoring posterior uveitis (inflammation) [287]–[289]. FAF utilises blue-light excitation to form a brightness map and it is found commercially in fundus cameras, confocal scanning laser ophthalmoscopes and ultra-widefield imaging devices [290]. FAF falls short due to its low signal strength and the tendency to produce autofluorescence artifacts, all whilst being potentially harmful to the retina and causing patients’ discomfort without device-specific mitigations [290]. These challenges have been overcome to achieve FAF systems capable of diagnosing and monitoring age-related macular degeneration, central serous chorioretinopathy, macular dystrophies and more, discussed in Yung *et al.*, but it does not so readily detect inner retinal (retinal ganglion cell) damage [290]. Malamos *et al.* utilised lipofuscin, the main source of autofluorescence in human fundus, in patients diagnosed with uveitis using FAF, demonstrating its potential as a non-invasive, single follow-up tool for progressive inflammatory disorders affecting the outer retina [291].

### D. Hyperspectral Imaging

Hyperspectral imaging (HSI) involves the acquisition of two-dimensional images across a broad range of the electromagnetic spectrum. The precise number of wavelengths varies in the literature and there is some overlap and often arbitrary differentiation from multispectral imaging (MSI). However, HSI is differentiated from MSI by primarily relying on the use of narrow adjacent spectral bands over a continuous range, as opposed to the discrete and spaced wavelength bands in MSI [292]. The number of bands can range from two up to several hundred. Target illumination is delivered by a white broadband light source *e.g.,* a halogen lamp, although supercontinuum lasers may also be used. The specific interactions of the light with the various chromophores in the tissue can result in reflected, emitted or fluorescent light from the sample, which are received by a detector [293], [294].

HSI can provide real-time images of a sample and as such has application in many tissues and biological contexts. Regarding the brain, it has been used to assess cerebral blood flow and tissue oxygenation [295], [296], and unlike NIRS, HSI is able to provide high resolution images and can potentially target multiple chromophores at a time. One drawback is that it is an invasive procedure requiring access to the target tissue, limiting its potential for *in-vivo* human applications, due to complications introduced by complex imaging equipment and computational issues [297]. However, there are a limited number of HSI applications for *in-vivo* brain metabolism and haemodynamic measurements, reviewed here [297], and it has successfully been used to image retinal vasculature [298]–[300]. Due to the close relationship of retinal and cerebral physiological states [299], the investigation of retinal vasculature with HSI in the context of TBI is promising [20].

Multispectral imaging has also been developed towards ophthalmic applications, although again these are focussed on the outer retina. Histological parameters have been extracted from multispectral images of the human ocular fundus to map the concentration and distribution of the retinal haemoglobins, choroidal haemoglobins, choroidal melanin, RPE melanin and macular pigment [301], [302]. Further work has completed in this field to obtain images of the human retina in rapid succession to eliminate discrepancies from saccades (natural eye movements) and reduce exposure times to 0.05s [303]. This exploration of multispectral imaging establishes it as viable as a quantitative analysis technique for the diagnosis of eye diseases like diabetic retinopathy and age-related macular degeneration (AMD) [303], and developments in novel multispectral analysis methods would reduce long computational times to aid the practicality of multispectral imaging being used in clinical settings [304].

### E. Near-Infrared Spectroscopy

Near-infrared spectroscopy (NIRS) is an *in-vivo* technique that has undergone considerable testing in human subjects [305]–[311]. It utilises wavelengths between 700 nm and 1000 nm, which can penetrate the skull and several millimetres into brain tissue, to monitor brain cortical perfusion and oxygenation. Attenuation of reflected light by differential chromophore absorption including oxygenated and deoxygenated haemoglobin and cytochrome-c-oxidase [312], enables their quantification in tissues, and determination of cerebral oxygenation changes as a ratio of oxy to deoxyhaemoglobin [313].

NIRS cannot discriminate between arterial, capillaries or venous blood and therefore provides a combined reading of tissue blood oxygenation. As venous blood is most abundant within the cranium, the normal cerebral oxygenation value obtained by NIRS is lower than that obtained by pulse oximetry which assess the pulsatile arterial blood oxygenation signal [314]. NIRS correlates with jugular venous blood oxygenation [315], which is indicative of the relationship between cerebral blood flow and cerebral metabolic and oxygen requirements [316]. Thus, where cerebral perfusion may be decreased by raised intracranial pressure or systemic blood volume loss, or cerebral tissue metabolic changes take place [317], NIRS enables the assessment of brain tissue oxygenation and cerebral perfusion, as well as the inference of changes in cerebral perfusion autoregulation and metabolic state during the early post-traumatic period.

Rodlan *et al.* investigate and review the promising applications of NIRS in TBI monitoring, collating examples of experiments that find good agreement between NIRS and current gold standard techniques, *i.e.,* neuroimaging, ICP monitoring [318]. They highlight that algorithms used within NIRS, wavelengths and source-detector separation differ between commercial systems, complicating direct comparisons and thus hindering the ability to characterise pathologies such as TBI severity. This challenge is exacerbated by extracerebral contamination introduced, the scalp, skull and CSF, and regional differences in pigmentation and pathophysiology [312].

### F. Terahertz Spectroscopy

Terahertz (THz) spectroscopy uses electromagnetic radiation in the wavelength range between microwaves and infrared to detect properties of matter. The vibrational and rotational energy of many biomolecules, such as proteins, are within this range thus making it an effective analytical technique that does not require pre-processing or labelling [319], [320]. The high sensitivity THz spectroscopy with the content and state of water in biological tissues has drawn interest in biophotonics [321], whilst also exhibiting non-invasive and non-ionizing characteristics [322]. Whilst it has been investigated in live animal models of TBI, successfully differentiating between traumatised and normal brain tissue [322], [323], it is used as an *ex-vivo* technique and requires tissue to be sectioned. Wang *et al.* identify THz spectroscopy as an early diagnostic tool for blast-induced TBI and able to differentiate the serum and CSF of mice with differing TBI severities [323]. Therefore, as a future clinical tool, THz spectroscopy would be much better suited for the analysis of liquid samples such as serum and CSF, rather than a POC diagnostic technique. However, THz spectroscopy is a relatively new biochemical imaging technique, and current limitations may be due to the lack of representation in literature. Further research efforts could identify THz as an ideal tool for rapid, label-free biological imaging.

### G. Raman Spectroscopy

**Fig. 7.**
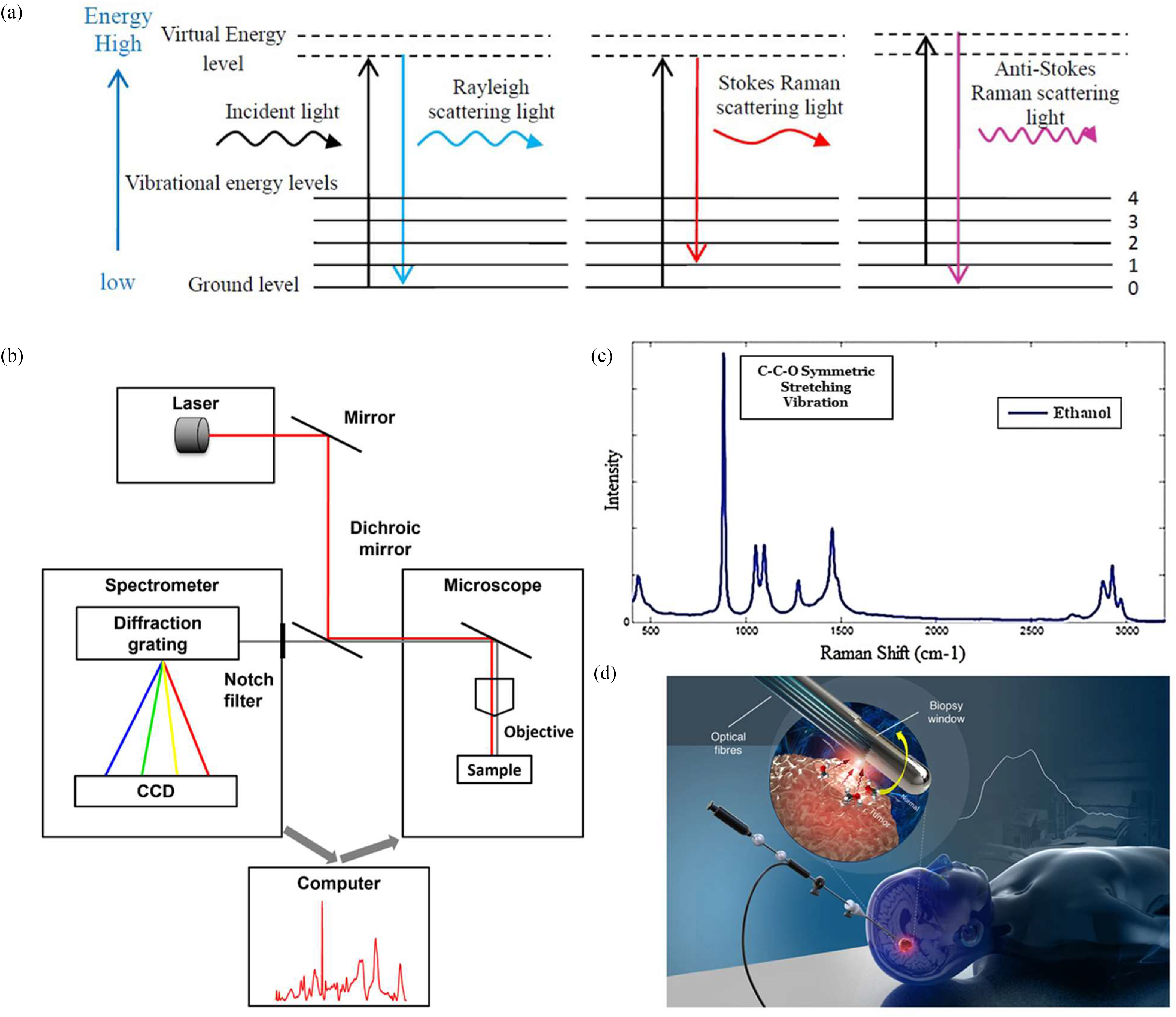
(a) Diagram of the energy transitions involved in Raman (inelastic) scattering compared to Rayleigh (elastic) scattering. In Stokes scattering, the incident photon has greater energy than the scattered photon, whereas the incident photon in anti-Stokes scattering has lower energy [123]. (b) Schematic diagram of a generic Raman Spectroscopy system, reproduced with permission from [330]. (c) Representative Raman spectrum of Ethanol with a prominent characteristic peak at 882cm^−1^ of the C-C-O bond symmetric stretching vibration [331]. (d) Schematic of a Raman fibre optic probe, an example of a popular RS development for clinical applications, used here for an optical core needle biopsy for in-vivo detection of brain cancer tissue [350].

RS is a powerful, sensitive and specific technique capable of measuring the chemical composition of complex samples, which can be accomplished non-invasively in a label-free manner [324], [325]. In this process, illustrated in Fig. 7(a), incident monochromatic coherent light provides identical packets of energy in the form of photons. In Raman scattering or inelastic scattering, incident photons are momentarily absorbed, setting the sample molecule into a bigger vibration, transitioning it to a higher excited, vibrational “virtual” state, resulting in the release of a new photon when the molecule falls from the virtual state back to a lower state [268], [326], [327], the scattered photons have a different energy from the incident ones, with a change equal to the energy required to vibrate the molecule to the higher excited state [328]. Each type of a molecular bond vibrates at a different frequency, requiring different amounts of energy for photons being scattered and thus, different frequencies hence, constituting of the Raman signal.

Given that the intensity of a Raman signal is a millionth of the incident light source due to the low probability of inelastic scattering [326], it is vital for the sufficiently sensitive RS system to be constructed using high-performing components to ensure minimal signal loss. A generic RS set-up is provided in Fig. 7(b) [329], the Raman signal is fed into a spectrometer where it can be detected and converted into a visual output as a spectrum of wavenumber (cm^−1^) against intensity (arbitrary units) [325].

A wavenumber is the spatial unit of frequency, indicating the difference between the energy and thus, wavelengths of the incident and scattered photons [326]. This results in multiple peaks that represent the vibrational modes that are characteristic for each molecule present in a given sample, creating a biochemical molecular fingerprint for a certain excitation wavelength [326]. A representative Raman spectrum of ethanol shows the characteristic peaks at 882cm^−1^ due to C-C-O bond symmetric stretching vibrations along with two peaks at 1050cm^−1^ and 1090 cm^−1^, due to the C-O bond scaling and C-C-O bond stretching vibrations, respectively (Fig. 7(c)) [330], [331].

The many attributes of RS have indicated a potential promise for its implementation in clinical settings, including its sensitivity in rapid molecular sensing and the unique capability to be employed outside the laboratory without significant loss of performance [190], [332]. Furthermore, for biological applications, where often a high-water content is present, RS, utilising visible or near-infrared light, enables highly resolvable *in-vivo* measurements with the signal from water being negligible due to the reduced absorption effects [324], [325].

In the past decade RS systems have become more portable, modular and more sensitive by utilising optical fibres, endoscopes, nanostructures and micro-spectroscopy [234], [332]–[334], making them more suitable for clinical and POC applications.

#### 1) Raman Spectroscopy in Neurodegeneration

Raman spectroscopy (RS) has shown promise in neuro-applications and has been utilised to analyse neurochemistry [335], [336], *in-vitro* neuronal cells [337], cancers [338]–[341], and cerebral brain metastases [332], [342]. Fibre optic Raman probes are handheld systems comprising an optical arm to deliver excitation light and a collection arm to detect the Raman signal [325], [333], [343]–[347]. There have been numerous fibre optic Raman probes developed for clinical applications, Fig. 7(d) features an example, that can diagnose numerous cancers *ex-vivo* using either biopsies or blood samples [348], as well as imaging intact brain tissue during a surgery [332], [348], [349], directly measuring the CSF obtained through existing external ventricular drainage devices [350].

RS also detected neurodegeneration, allowing early and sensitive neurodiagnostics through the rapid, non-invasive spectroscopic assessment of biofluids at the point-of-injury [168]. Most current successful RS systems are geared towards *ex-vivo* characterisation of ND diseases, focussed on the study of blood and tissue in both animal and human models, with only a single human study of MS, in which Bergholt *et al.* investigated the lipid content of post-mortem brain tissue [351]. Further studies employed CARS in murine MS models [352]–[354], and on tadpoles using surface enhanced Raman spectroscopy (SERS), showing the *in-vivo* capability for real-time imaging of an MS model [355], highlighting the non-destructive nature of RS for research into neurological diseases.

RS applications in AD diseases are more extensive and the research is majorly split into the characterisation of CSF or blood to identify protein and lipid biomarkers [326]. The tracking of blood biomarkers has been investigated with SERS and CARS to allow for very low concentrations of tau protein and amyloid-beta to be monitored [356]–[361]. RS also allowed sensitive AD detection using SERS, detecting trace level amyloid-beta [344], [362], [363]. Although, most studies concluded that further larger scale clinical validation and optimisation was required for successful transition into a clinic, they highlighted the rapidness, sensitivity and specificity achievable using the various RS methods, successfully measuring various neurological molecular changes in patients.

#### 2) Raman Spectroscopy in TBI

RS is a non-invasive technique able to measure biomarkers rapidly and in real-time. As a molecular sensing technique, RS could be a powerful method for detecting TBI indicative biomarkers [190], particularly given the significant cascade of biological and chemical metabolic changes accompanying TBI, which could provide diagnostic information in the acute phase and detect the development of secondary injuries [61], [225]. User-friendly, hand-held POC devices capable of monitoring a panel of TBI biomarkers, using miniaturised RS hold the biggest promise for TBI diagnosis [175], [268], [327], [341], [364], [365].

The multiplex ability to obtain chemical information and detect low-level biochemical changes in tissue samples makes it a potentially powerful technique in, not only diagnosing TBI but also in, deciphering between mild, moderate, and severe cases. RS applied in animal TBI injury models [366]–[368], and human plasma [234], [369], demonstrates the feasibility of spectroscopically identifying and classifying head injury and its severity, and how RS can monitor changes in tissue biochemistry after trauma [366], [367].

TBI indicative biomarkers from human plasma have been characterised using surface enhanced Raman spectroscopy (SERS). One system demonstrates that combining an optofluidic, SERS lab-on-a-chip with a portable RS system presents promise for injury diagnostics and subsequent monitoring by profiling the levels of the NAA, S100B and GFAP [234]. Another system incorporates portable SERS into a disposable paper-based lateral flow strip to detect NSE in blood plasma samples [370]. Both methods provide alternatives to commonplace ELISA approaches that can be deployed rapidly, portably and with small sample sizes, ideal parameters for POC settings. SERS has also been utilised for *ex-vivo* studies in detecting and monitoring TBI biomarkers in various biofluids [371], [372].

## V. Raman Spectroscopy and the Eye

### A. Ex-Vivo

RS analysis of tears and tear meibum have shown promise, studying composition [373]–[378], eye-related disease [376], [379]–[381], non-eye-related disease [382], [383] and contact lens use [384]. RS of tears has also been used for assessments of AD and mild cognitive impairment using principal component analysis (PCA), to detect change in healthy and disease states [374]. RS can monitor changes due to surgery, therapy and age [385]–[388]. In addition, resonance RS (RRS) determined the efficacy of a scleral iontophoresis device (drug delivery through a gradient) by measuring the levels of lutein in the sclera, choroid, retinal periphery and macula following therapy [387], and analysed age-related changes in *ex-vivo* Bruch's membrane samples, the inner-most layer of the choroid, suggesting the potential of doing the same *in-vivo* to determine those at high risk of ocular disease and the success of therapy and also that Bruch's membrane and sclera are composed of similar biomolecules, suggesting the sclera could be used as a viable (and more readily accessible) surrogate marker for changes in the Bruch's membrane [388], [389].

*Ex-vivo* studies of enucleated human eyes have shown promise for development of ocular RS [387], [390]–[396]. The retina has a dense neuron structure and it is a highly metabolically active tissue [114], [397], which can be accessed externally through the optics system of the eye to perform RS. A method to measure carotenoid levels in flat-mounted retinae, was an initial proof-of-concept for future *in-vivo* studies [398], [399], using carotenoids (a biomarker for macular pigment level), detecting lutein and zeaxanthin resonance enhancement when excited by a 488 nm excitation wavelength, which has been widely replicated [385], [387], [392], [400]–[402]. The presence of validated TBI biomarkers in the eye such as elevated concentrations of tau, (detected using immunohistochemistry), in the retina supports the potential of TBI as an ocular diagnostic tool [123].

**Fig. 8.**
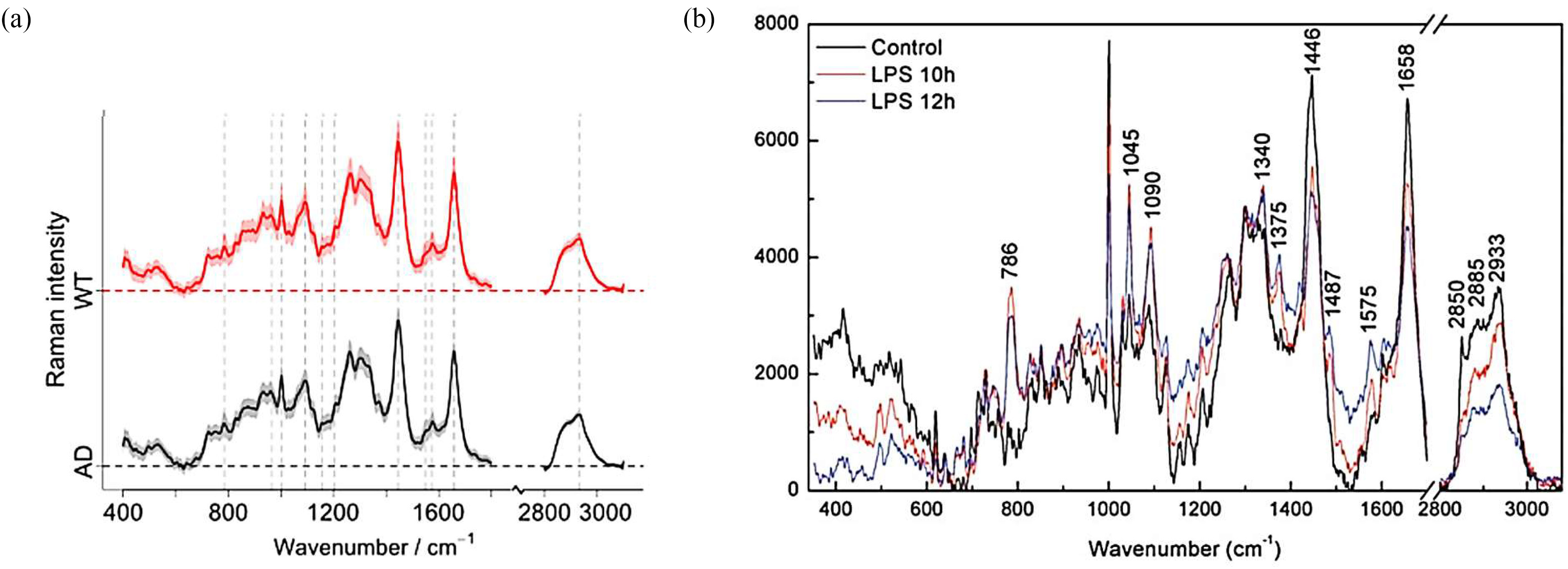
Raman spectra of eye tissue undergoing chemical changes indicative of neurodegeneration. (a) Raman spectra of en-face murine retina using 785 nm laser, grouped into wild mice and AD model mice. Chemometric analysis revealed biochemical changes indicative of structural and pathological manifestations of AD, reproduced with permission from [144]. (b) Spectra of murine, retinal cultures modelling MS using LPS, measured using a 785 nm Raman system. Increasing incubation with LPS lead to changes in the heights of characteristic peaks in the spectra, indicative of neuroinflammation [404].

Stiebing *et al.* continued previous work in mouse retinae, identifying retinal layers using RS and discriminating between healthy and AD subjects [144]. They found that spectra obtained of *en-face* healthy and AD retinae are not distinguishable without statistical analysis to detect underlying changes (Fig. 8(a)), which was able to correctly recognise AD retinae with a sensitivity of 86.2% [144]. The inclusion of *en-face* samples, as well as probing the retinal layers, mimics *in-vivo* measurements which makes the study more translatable once an *in-vivo* system has been achieved. Marro *et al.* studied *in-vitro* murine retinal cultures treated with lipopolysaccharide to create an MS model of the retinal ganglion layer (RGC) using an *in-vivo* RS measurements of the tissue, examining neuroinflammatory molecular changes as a function of time [403]. Subsequently, curve fitting with known retina components was utilised to de-convolve the Raman spectra highlighting bands which undergo changes with increasing LPS treatment time (Fig. 8(b)) [403]. The authors identified markers in the molecular components of the RGC layer which indicate changes in inflammatory mediators, components of mitochondria and fatty acids during neuroinflammation.

### B. In-Vivo RS in the Eye

*In-vivo* RS measurements of the lens has also been explored with the aim to identify the presence of artificial intraocular lenses used for the treatment of cataracts and investigated the effect of lens yellowing and transparency loss on *in-vivo* RS measurements [402], [404]. In post-mortem lenses of 7 dementia patients with confirmed diagnoses of AD high lens amyloid-beta levels did not correlate with brain tissue immunostaining, although the research demonstrated that RS could take non-destructive measurements in the eye [405]. Martinez-Lapiscina *et al.* explored the prospect of monitoring inner nuclear layer thickening within the retina by coupling RS with a confocal scanning laser ophthalmoscope. The study measured key molecules of inflammation and neurodegeneration in human retina, demonstrating the potential of RS non-invasively addressing molecular changes of *in-vivo* CNS *via* the retina [146].

In 1998, Gellermann *et al.* provided a proof-of-concept design that used RRS to obtain *in-vivo* macular pigment measurements within the human retina, patented in 1999, limiting the majority of succeeding publications to the same research group and method [406]–[411]. The macular pigment has been investigated this way to monitor macular pigment levels decreasing with decreasing foveal thickness following surgery [385], [412], to identify a 50% decrease in macular carotenoid pigments in patients with Stargardt macular dystrophy compared to healthy controls [413], to examine macular pigment optical density to determine macular pigment level decrease following intraocular lens implant [386], [414], and the intake of lutein supplements [415].

**Fig. 9.**
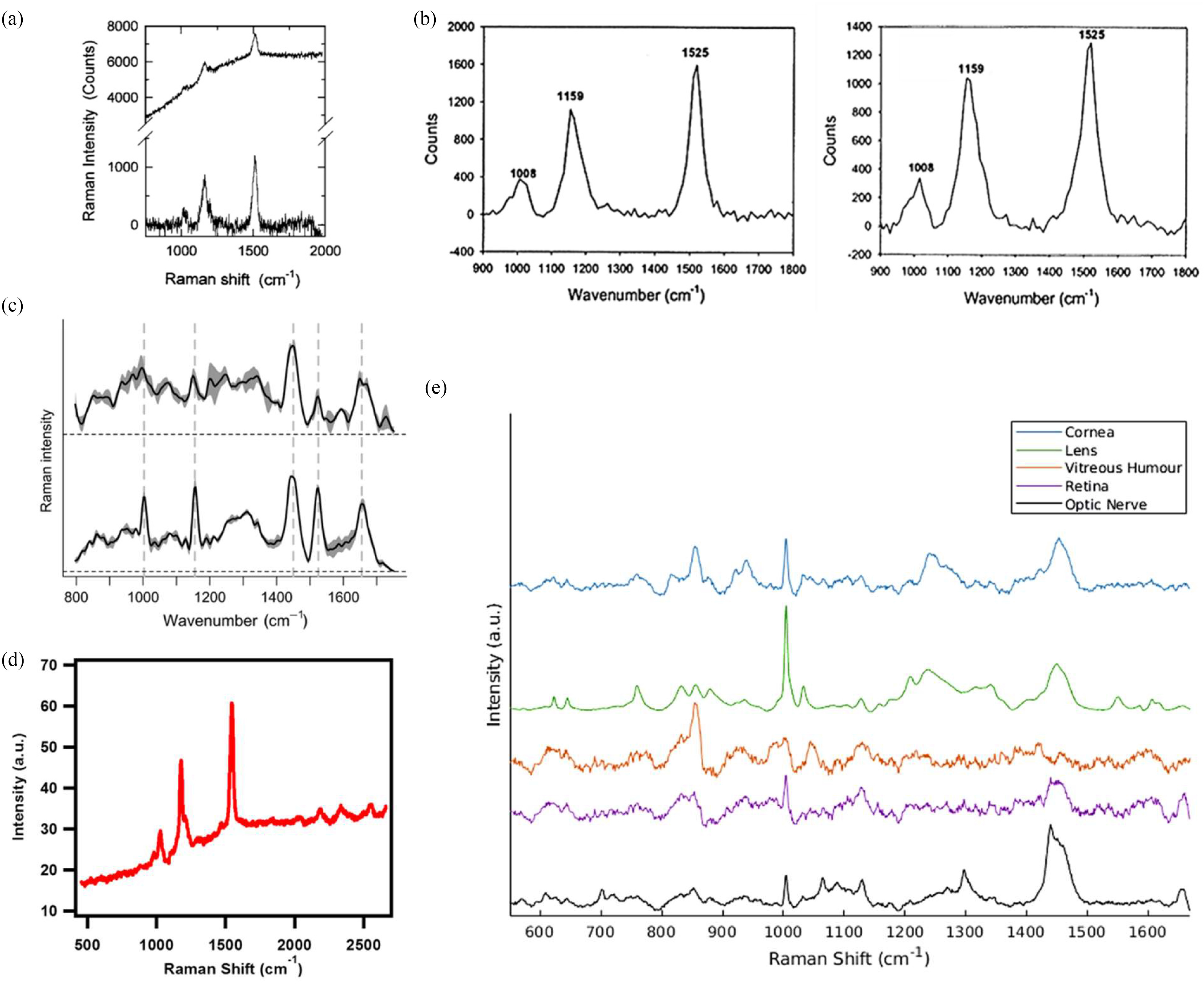
Raman spectra of in-vivo samples and models which simulate an in-vivo environment. (a) Raman spectrum of healthy, human retina, measured in-vivo with dilated pupil (∼8 mm diameter), using a 488 nm laser. The top spectrum is from 3 summed measurements and the bottom spectrum is the same measurements with baseline subtracted. Characteristic carotenoid peaks are present at 1008, 1159 and 1525 cm^−1^, reproduced with permission from [411]. (b) Comparative study of Raman spectra taken in a human eye in-vivo and macular carotenoid zeaxanthin in liquid form within an eye model, reproduced with permission from [417]. (c) Comparative spectra of flat-mounted, murine retina using 785 nm lasers within a commercial Raman system (bottom) and an in-house built Raman set-up (top) which simulates in-vivo parameters in the eye. Characteristic carotenoid peaks are present in both [422]. (d) Spectra taken of ex-vivo macular pigment tissue samples which were fixed using formalin fixative, using a 488 nm excitation wavelength [393]. (a)–(d) demonstrate that the same characteristic carotenoid peaks are present in in-vivo, fresh ex-vivo and fixed ex-vivo eye samples. (e) Raman spectra of fresh, ex-vivo, porcine eyes that were dissected into 5 main features. Measurements were taken using a commercial system, 785 nm laser and settings were chosen based on the maximum permissible exposure defined by eye-safe limits [367].

Resonant Raman spectroscopy (RRS) with a 488 nm excitation wavelength to create a strong vibrational response from the carotenoid, permitted a lower laser power and exposure time to be used compared to previous work and facilitated confocal Raman spectra of an *in-vivo* human retina, measuring the concentration of macular carotenoid pigments lutein and zeaxanthin (Fig. 9(a)) and obtaining clear carotenoid peaks [409]. In 2004, the authors provided an update regarding the method obtaining spectra using eye-safe exposure levels in just 0.25 second and its use in clinical trials [410]. That same year, the group corroborated their results by taking spectra of macular carotenoids through a “model eye” to mimic *in-vivo* conditions, obtaining clear results (Fig. 9(b)) [416]. More recently, the same research group has investigated the use of photometry [417], which has been explored in other literature to validate the reproducibility of Bernstein's method [385], [401], and have been able to distinguish lutein and zeaxanthin within *ex-vivo* retina samples, measuring macular pigment distribution using Raman spectroscopy and autofluorescence imaging [418]–[420].

With eye-safe RS laser safety protocols, protein, lipid and nucleic acid were detected on mounted retina tissue samples, *ex-vivo* using the 785 nm excitation wavelength predicting the success of the future *in-vivo* RS system to diagnose macular degeneration [421]. The study compares spectra from an in-house built RS set-up to those obtained by an optimal confocal Raman imaging system (Fig. 9(c)), finding good agreements and predicting the success of the in-house built system to diagnose macular degeneration *in-vivo* [421]. Banbury *et al.* have recently detected, distinguished and categorised the response of segments of an *ex-vivo* porcine eye samples using a 785 nm excitation wavelength (Fig. 9(e)) [366]. If similar results could be achieved using near-IR wavelengths, to reduce tissue auto-fluorescence [325], whilst adhering to eye safety regulations, these studies lay the platform for potential developments of non-invasive, *in-vivo* RS systems for real-time diagnostics and monitoring of TBI.

## VI. Towards Ocular Raman Spectroscopy for TBI Diagnostics

Detection of biochemical ocular TBI biomarkers, requires *in-vivo* and eye-safe molecular sensing techniques that can characterise the chemical constituents of the eye, suggesting RS as a potential solution. RS using an 800 nm excitation laser was used to detect glutamate in whole *ex-vivo* porcine eyes as well as to characterise the various eye segments [422]. In ocular diseases such as AMD, glaucoma, diabetic retinopathy and retinal detachment, increased levels of glutamate in the retinal nerve cells diffuse into neighbouring tissue suggesting that detection of glutamate biomarker excitotoxic neuronal death [423], which indicates the potential of spectroscopic integration of TBI indicative biomarkers *via* the eye. Banbury *et al.* have recently demonstrated exploitation of eye-safe RS to measure the biochemical response in the retina of an *ex-vivo* murine TBI tissue, using a 633 nm excitation wavelength. The acquired data was subsequently analysed using advanced machine learning algorithms, successfully classifying injury severity levels of severe, moderate and control cohorts with sensitivities of 82.0%, 75.1% and 69.4%, respectively [424]. Recently, the high wavenumber peaks from fatty tissue samples, similar to that found in the brain were successfully measured using a non-invasive portable Raman spectroscopy device comprised of a FUNDUS camera and an un-dilated eye phantom, laying the platform for the *in-vivo* retinal measurements for TBI diagnostics at the POC. RS applied to murine retina organotypic cultures undergoing an inflammatory response, creating a spectral library of 5 biomolecules which yielded the strongest peaks in response to neuroinflammation, including NAA and glutamate [425], [426]. Both studies demonstrated the effectiveness of RS with PCA statistical analysis to detect and monitor neuroinflammation in the retina and potentially identifying retinal metabolites present in ocular manifestations of TBI.

For the immediate future, there is a need to validate recent findings through internal and external references. Internal references refer to a method of direct analysis of brain tissue *in-vivo* in humans in the context of TBI. We have recently performed a preliminary work for incorporating Raman spectroscopy into an existing standard of care for invasive monitoring in intensive care following TBI, *via* an external ventricular drainage device [427]. This approach reduces the barrier to entry for ethical approval and allows ground truth assessment with respect to the GCS and ICP. External referencing refers to validation of the biochemical attributions made by a means other than Raman spectroscopy. Since it is possible to measure the ON sheath diameter using MRI [136], it may also be feasible to measure chemical information from the ON sheath using MRS. Chemical species detected by MRS such as, the S100B, GFAP or the NAA could then be used as reference compounds in a fitting library for complementary Raman spectra. However, this requires further developments of the portable Raman devices and ethical approval for use *in-vivo*.

RS can offer a label free mechanism for measuring changes to biochemistry, which can be applied *in-vivo* in invasive settings such as surgery but has also shown promise for non-invasive measurement, in the field of ophthalmology. Given a rooting in more fundamental chemistry, the analysis of spectra from biological samples, formed of complex permutations of thousands of individual molecules in a single sample remains challenging [332], [428]. Although RS has proven to be employable outside of laboratory settings, Raman signals are intrinsically weak and clinical applications introduce additional factors including the surgical lighting, excess blood, unstable patients and the complexity of living tissue [332]. Historical chemometric analysis tools such as, PCA are commonly used inappropriately in the literature in efforts to overcome the high dimensionality of the data. By aiming to study posterior tissue through a thick heterogeneous sample (the eye) and indirectly detect subtle changes from brain injury, will require improvements to analysis methods. The link between damage to the retina and CNS has been noted in the literature [23], [38], [99], but this has not currently been applied to a specific model of disease or injury. In exploring whether it is fundamentally possible to study biochemical changes resultant from TBI will expand the possibilities of RS as an emerging diagnostic platform.

## VII. Summary and Prospects

TBI is a silent, world-wide epidemic, affecting populations of all ages, in civilian and military life, in work, home and sporting activities with significant long-term morbidity and economic impacts. Current POC triage and diagnostic tools do not reliably allow timely intervention (under-triage) and often waste resource through over-triage. There is therefore a strong need for reliable, early, POC molecular diagnostics to support triage and clinical decision-making to allow patient treatment to improve outcomes within the golden hour.

This paper has presented an overview of the status, opportunities, and obstacles faced as researchers begin to explore early diagnostic tests for TBI and other neurodegenerative diseases. The translation of neurological biomarkers from the bench to the secondary care and pre-hospital, POC arenas is progressing rapidly in a number of areas, showing promise particularly in spectroscopic techniques such as RS. To accelerate the field of biomarker imaging, categorisation is key. Clear standard ranges and concentration thresholds of TBI biomarker(s) in blood and CSF the acute phase after injury would accelerate diagnoses and severity characterisation for all biomarker detection techniques. Not only would this facilitate diagnoses but also aid better understanding of an incredibly heterogeneous pathology, stratifying biochemical responses to differing injury severities and guiding potential therapies. Raman spectroscopy therefore has the potential to provide complete non-invasive imaging through the eye. Employing this technique in a portable system would mitigate limitations and ambiguities introduced by the GCS and triage patients during the critical time before transport to emergency departments. In time-sensitive and remote settings such as in military service or at roadside or sport pitch-side settings, this would monitor ongoing health to protect patients from secondary or repeat injury and therefore long-term neurological, cognitive and psychological morbidity.

TBI diagnostics is a broad field with clear objectives to improve the speed and precision of diagnostic techniques to maximise patients’ neurological recovery. However, there are few therapies mentioned when discussing medical intervention following diagnoses, another great unmet need. Advances in TBI treatments would work in synergy alongside developments in diagnostic techniques to improve the patient journey and reduce deaths from TBI worldwide. All available technologies have challenges relating to specificity and sensitivity, but the potential of RS and other techniques as new additions to the ocular and TBI diagnostics toolbox is significant and several ongoing research efforts in TBI should start to be realized in the coming years.

*Disclosure Statement:* The authors declare no conflict of interest.
